# Novel cancer‐associated secretory cells and IL‐1β^+^ macrophages as key players in early lung adenocarcinoma progression in female never‐smokers

**DOI:** 10.1002/ctm2.70433

**Published:** 2025-08-18

**Authors:** Junsu Choe, Jinyeong Lim, Noeul Kang, Eun Seop Seo, Mina Hwang, Kyung Yeon Han, Se‐Hoon Lee, Myung‐Ju Ahn, Yoon‐La Choi, Hong Kwan Kim, Woong‐Yang Park, Sang‐Won Um

**Affiliations:** ^1^ Division of Pulmonary and Critical Care Medicine, Department of Medicine Samsung Medical Center, Sungkyunkwan University School of Medicine Seoul Republic of Korea; ^2^ Department of Health Sciences and Technology SAIHST, Sungkyunkwan University Seoul Republic of Korea; ^3^ Division of Allergy, Department of Medicine, Samsung Medical Center Sungkyunkwan University School of Medicine Seoul Republic of Korea; ^4^ Samsung Genome Institute, Samsung Medical Center Seoul Republic of Korea; ^5^ Division of Hematology and Oncology, Department of Medicine, Samsung Medical Center Sungkyunkwan University School of Medicine Seoul Republic of Korea; ^6^ Department of Pathology and Translational Genomics Samsung Medical Center, Sungkyunkwan University School of Medicine Seoul Republic of Korea; ^7^ Department of Thoracic and Cardiovascular Surgery, Samsung Medical Center Sungkyunkwan University School of Medicine Seoul Republic of Korea

1

Dear Editor,

We identified novel cancer‐associated secretory (CAS) cells and interleukin (IL)‐1β^+^ macrophages as key contributors to the progression of lung adenocarcinoma (LUAD) exhibiting part‐solid radiological features in female never‐smokers.

On radiologic‒pathologic correlation, the ground‐glass (GG) and solid (S) components on CT imaging corresponded to lepidic and invasive histological patterns, respectively. Early‐stage part‐solid LUADs predominantly harbour epidermal growth factor receptor (EGFR) mutations and exhibit indolent growth patterns, making them ideal models for studying early tumourigenesis with minimal confounding from smoking‐related factors. However, the key genomic alterations driving this progression remain poorly understood.^1^ We performed a multiomics analysis of four spatial components—S, GG, adjacent normal (AN) and distant normal (DN) lung tissues—from part‐solid‐type LUADs in 11 female never‐smokers with stage I disease. Analyses included whole‐exome sequencing (WES) and whole‐transcriptome sequencing (WTS) (*n* = 7), single‐cell RNA sequencing (*n* = 4) and spatial transcriptomics (*n* = 1, SMC‐19) (Figure S1A). Tissues were obtained during surgery under institutional review board‐approved protocols at Samsung Medical Center (no. 2017‐10‐022).

Patient characteristics and an overview are shown in Tables S1 and S2 and Figure S1A. WES showed EGFR sensitising mutations in 10 patients and a rare p.P772_H773dup in one, after filtering germline variants using matched blood and public databases. GG and S components displayed similar mutational profiles, including shared EGFR, RBM10, MTOR and TP53 mutations (Figure S1B). Transcriptomic analysis revealed increased glycolysis and cell cycle‐related pathways in both tumour components compared to the DN component, and upregulation of epithelial‒mesenchymal transition (EMT) and hypoxia pathways in the S component (Figure S1C,D).

Single‐cell RNA sequencing, incorporating 11 468 cells from two never‐smokers with advanced‐stage LUAD (AD), revealed dynamic shifts across DN, AN, GG, S and AD tissues (Figure S2).^2^ Myeloid, natural killer (NK) and endothelial cells decreased, while B and T cells increased from the DN component to AD. T and NK cell profiling revealed decreased cytotoxic CD8+ T, NK and adaptive NK cells, alongside increased naïve, central memory and regulatory CD4+ T cells, as well as effector memory CD8+ T cells towards the GG and S components (Figure S3).

The transcriptional features of epithelial cell types are presented in Figure 1A. We identified a CAS cluster, distinct from alveolar type 2 (AT2) and club cells, defined by high secretoglobin family 3A member 2 (SCGB3A2) and carcinoembryonic antigen‐related cell adhesion molecule 6 (CEACAM6) and minimal secretoglobin family 1A member 1 (SCGB1A1) expression; differentially expressed gene (DEG) analyses comparing CAS with AT2 cells—putative LUAD precursors involved in alveolar repair—and club cells further highlighted their molecular distinctiveness (Figure 1D and Tables S3 and S4). CAS cells expressed cancer‐related genes such as serine peptidase inhibitor Kazal type 1 (SPINK1), CXCL14, carcinoembryonic antigen‐related cell adhesion molecule 5 (CEACAM5) and CEACAM6. CAS cells exhibited a significantly increasing trend from the DN to the S component (*p* < .01) (Figure 1E).

**FIGURE 1:**
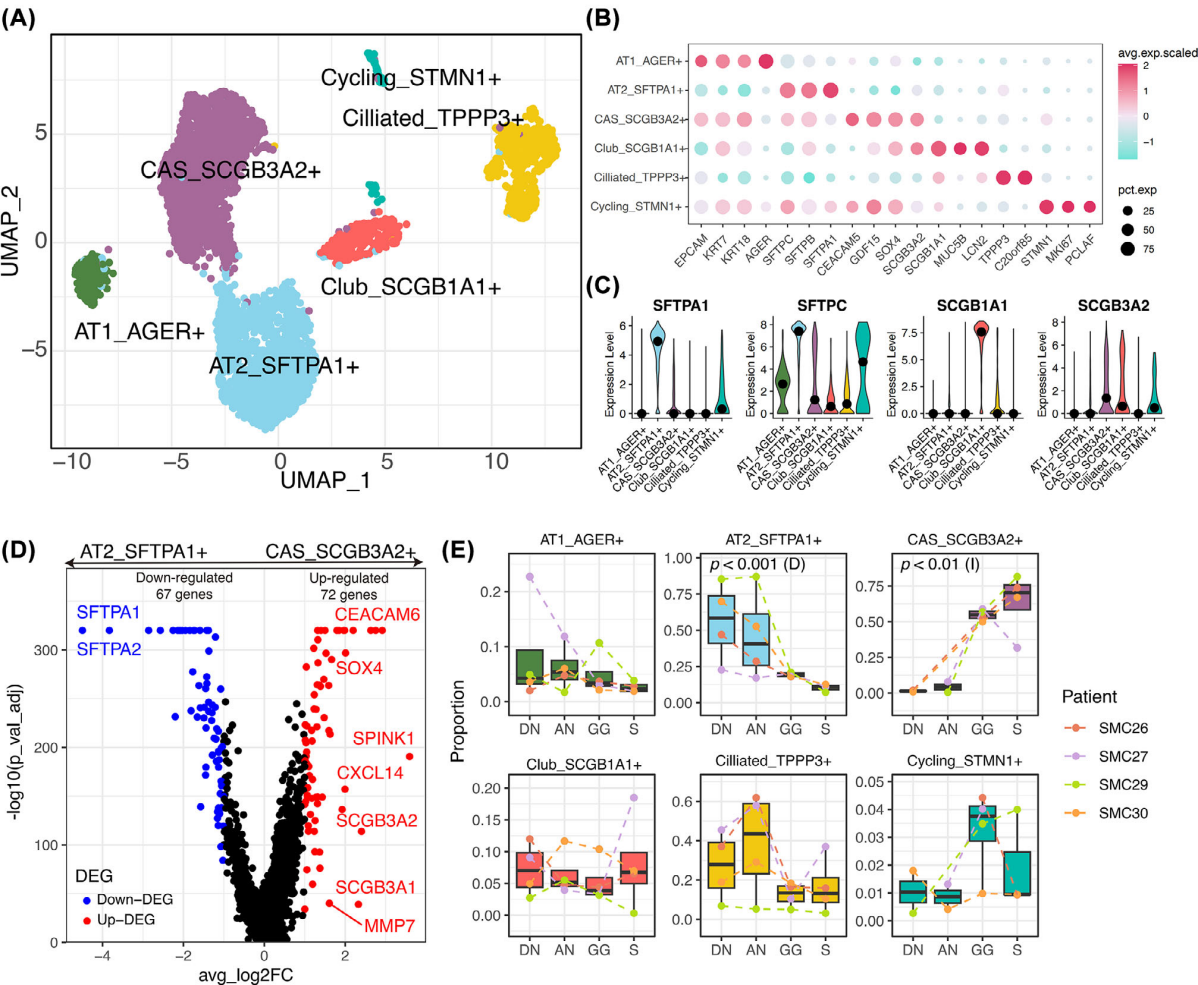
Identification of cancer‐associated secretory (CAS) cells and dynamic epithelial cell proportions across the four components of part‐solid‐type lung adenocarcinoma (LUAD). (A) Uniform manifold approximation and projection (UMAP) plot of 4691 epithelial cells from part‐solid‐type LUAD. Each dot represents a single cell, coloured according to cell type. (B) Feature plots of canonical markers used to annotate each epithelial cell subtype in the UMAP plots. (C) Violin plots showing the expression levels of advanced glycation end‐product specific receptor (AGER), surfactant protein A1 (SFTPA1), secretoglobin family 1A member 1 (SCGB1A1) and secretoglobin family 3A member 2 (SCGB3A2) cells across epithelial cell subtypes. (D) Volcano plot showing DEGs between CAS (SCGB3A2+) and alveolar type 2 (AT2) (SFTPA1+) cells. Thresholds were set at  .05 for adjusted *p*‐values (p_val_adj) and  .1 for average log fold change (avg_logFC). The maximum value of ‒log10 (p_adj) was 320. (E) Boxplot showing the proportion of each epithelial cell subtype across distant normal lung tissue (DN), adjacent normal lung tissue (AN), ground‐glass component of tumour (GG) and solid component of tumour (S). The colour of points and lines indicates patient‐specific data (*n* = 4). Jonckheere–Terpstra tests were performed across the four components, where (D) indicates a decreasing trend and (I) indicates an increasing trend.

We performed trajectory analysis using Slingshot to trace CAS cell origins. This analysis identified two main lineages in LUADs (Figure 2A). One lineage, represented by the black line, progressed from AT2 to alveolar type 1 (AT1) cells, reflecting normal lung epithelial cell development. Conversely, the second lineage, shown by the red line, is associated with tumourigenesis, primarily culminating in the S component. CAS cell projections varied across patients (Figure 2B). Lineage tracing showed early lineage 2 cells expressed the AT2 marker surfactant protein A1 (SFTPA1), then upregulated CEACAM6 while retaining SCGB3A2 (Figure 2C). Variability in SPINK1 and CEACAM5 expression further supported transcriptional heterogeneity (Figure 2D).

**FIGURE 2:**
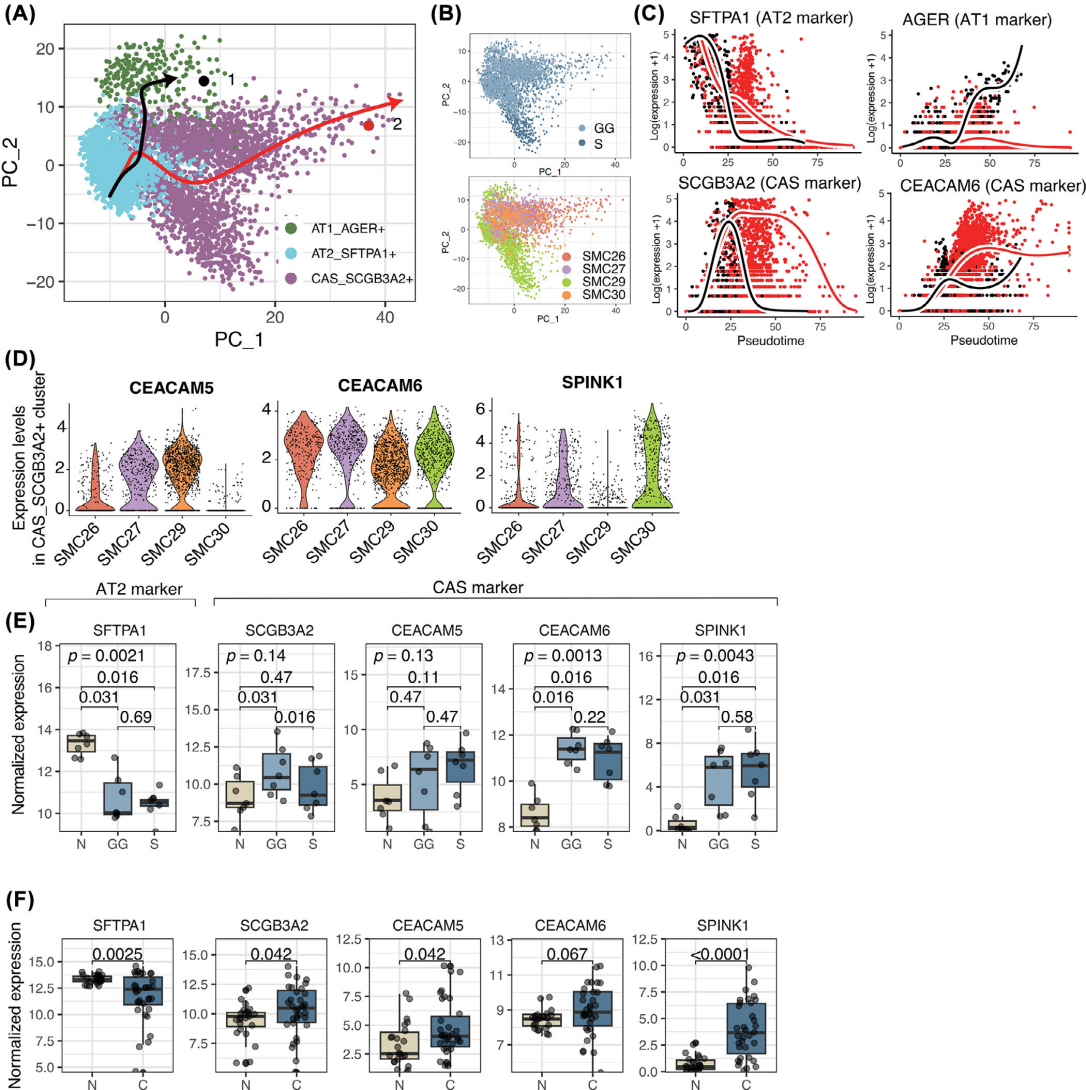
Cancer‐associated secretory (CAS) cells originated from alveolar type 2 (AT2) cells. (A) Principal component analysis (PCA) plot of alveolar type 1 (AT1), AT2 and CAS cells in the solid component of tumour (S) and ground‐glass component of tumour (GG) regions, with lines representing the inferred trajectories. Each dot represents a single cell and is coloured according to cell type. Lines indicate inferred trajectories, estimated using Slingshot. (B) PCA plots of single‐cell transcriptomes, with cells (dots) coloured by region (GG vs. S) (top) and patient (bottom). (C) Pseudotime analysis depicting the gene expression dynamics of surfactant protein A1 (SFTPA1) (AT2 marker), advanced glycation end‐product specific receptor (AGER) (AT1 marker), secretoglobin family 3A member 2 (SCGB3A2) (CAS marker) and carcinoembryonic antigen‐related cell adhesion molecule 6 (CEACAM6) (CAS marker) along the inferred trajectory. The black line and points represent lineage 1 (AT2 to AT1), while the red line and points represent lineage 2 (AT2 to CAS). (D) Violin plots showing the expression levels of carcinoembryonic antigen‐related cell adhesion molecule 5 (CEACAM5), CEACAM6 and serine peptidase inhibitor Kazal type 1 (SPINK1) across different samples in CAS cell types from single‐cell RNA sequencing (scRNA‐seq). (E) Box plots displaying normalised expression levels of SFTPA1, SCGB3A2, CEACAM5, CEACAM6 and SPINK1 across different components (N, GG and S, *n* = 7, respectively) in whole‐transcriptome sequencing analysis. The Kruskal–Wallis test was performed. (F) Box plots showing the normalised expression levels of SFTPA1, SCGB3A2, CEACAM5, CEACAM6 and SPINK1 across normal (N, *n* = 23) and cancer (C, *n* = 34) tissues from a study by Zhang et al. (2020). Wilcox statistical significance is indicated by *p*‐values. PSN, part‐solid nodule; SCGB3A1, secretoglobin family 3A member 1.

We identified the CAS cell type in seven patients who underwent WTS. A decrease in SFTPA1 expression (an AT2 marker) was observed, whereas CEACAM6 and SPINK1 (CAS markers) exhibited high expression levels in tumour regions (GG and S) compared to the DN region (Figure 2E). These patterns were consistent with Zhang et al., who reported similar marker expression differences between cancer and normal lung regions (Figure 2F).^3^

Sub‐clustering of myeloid cells identified four macrophage, one neutrophil and five dendritic cell subtypes (Figures 3A,B and S4). IL‐1β^+^ macrophages showed a pro‐inflammatory, M1‐dominant gene signature (Figure 3C) and expressed elevated levels of tumour necrosis factor‐alpha (Figure 3D), a key mediator of the proinflammatory response to pathogens. IL‐1β⁺ macrophages also expressed other pro‐inflammatory mediators (CXCL2, IL‐18, IL‐6; Figure 3D) and were associated with the nucleotide‐binding and oligomerisation domain (NOD)‐like receptor signalling pathway—a stress‐induced innate immune pathway (Figure 3E). IL‐1β^+^ macrophages (*p* = .01) exhibited significant increasing trends from DN to AN, GG and S components (Figure 3F).

**FIGURE 3:**
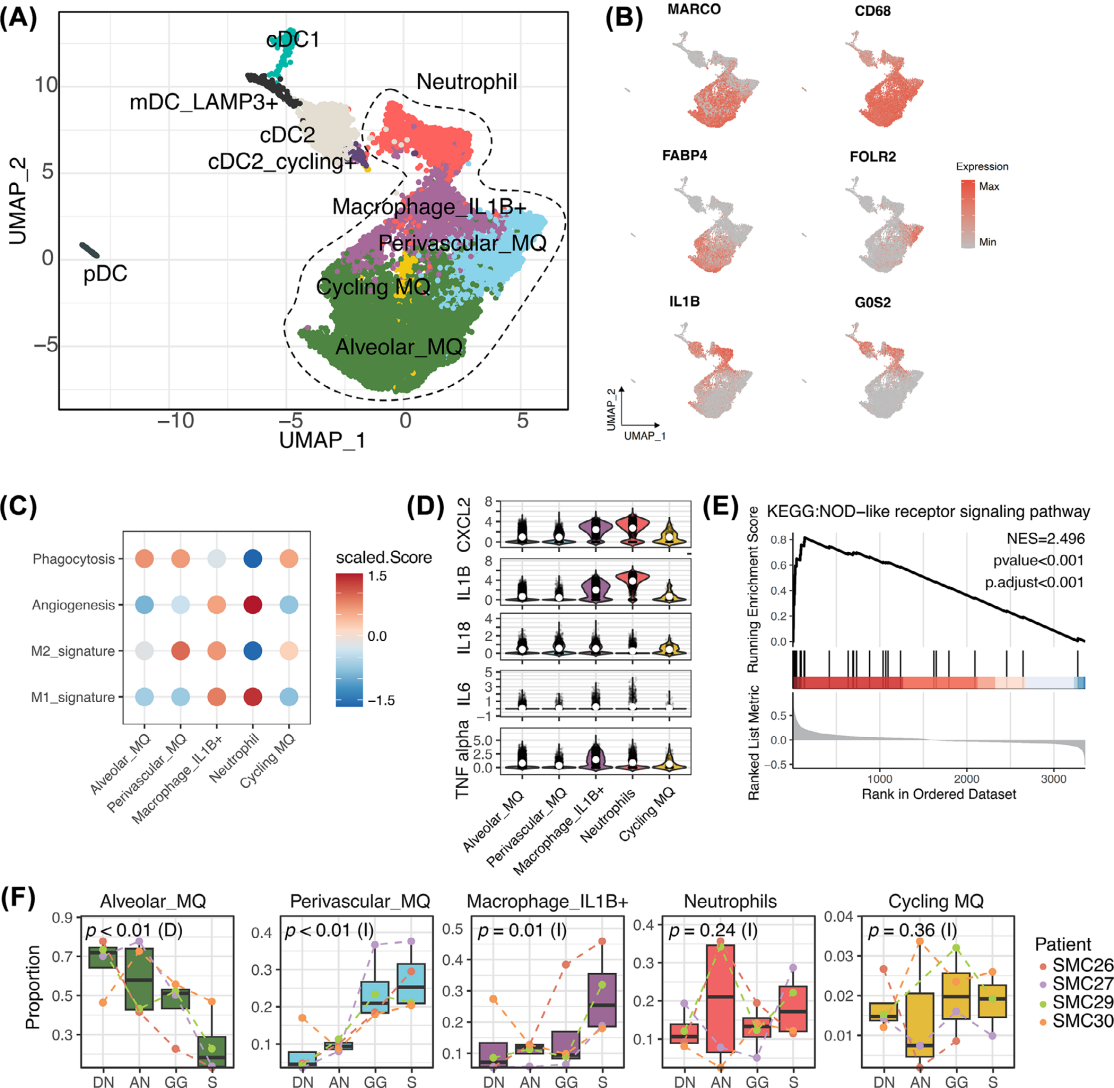
Composition and characterisation of the myeloid subsets in four components of part‐solid‐type lung adenocarcinoma (LUAD). (A) Uniform manifold approximation and projection (UMAP) plot of 14 143 myeloid cells revealing 14 subtypes. Each dot represents a single cell and is coloured according to cell type. (B) Expression of marker genes for the myeloid cell subtypes. (C) Dot plot showing the expression of macrophage‐related signatures, M1 signature genes, M2 signature genes, and phagocytosis and angiogenesis state scores. The colour represents the relative score. (D) Violin plot of gene expression in subsets of macrophages, comparing the proportion of myeloid cell subtypes. White dots indicate median values, while black small dots represent the expression levels of individual cells. (E) Gene set enrichment analysis (GSEA) analysis of the nucleotide oligomerisation domain (NOD)‐like receptor signalling pathway from the Kyoto Encyclopedia of Genes and Genomes (KEGG) database, using ranked differential expression gene lists of interleukin 1 beta (IL‐1β^+^) macrophages compared to other macrophage subtypes. (F) Box plots showing the proportion of myeloid cell subtypes across distant normal lung (DN), adjacent normal lung (AN), ground‐glass component of tumour (GG) and solid component of tumour (S). The colour of points and lines represents patient‐specific data (*n* = 4). Jonckheere–Terpstra tests were performed across the four components, with (D) indicating a decreasing trend and (I) indicating an increasing trend. GGN, ground‐glass nodule.

We performed spatial transcriptomics on an early‐stage LUAD (SMC‐19) to analyse spatial organisation of cell types in the solid and GG components (Table S2). Three major clusters were identified: invasive, lepidic, and bronchial areas (Figure 4A). Pathway analysis revealed activation of oncogenic and hypoxia‐related pathways in the invasive area (Figure 4B). A distinct region with heightened EMT and hypoxia signatures was also noted in the invasive area compared with the lepidic area (Figure 4C). Notably, CAS cells were concentrated in specific invasive regions co‐localised with IL‐1β^+^ macrophages (Figure 4D, red arrow). IL‐1β^+^ macrophage probability scores were positively associated with CAS/AT2 ratios, hypoxia and EMT signatures (Figure 4E,F). Ligand–receptor analysis in tumour regions (SS and G) identified prostaglandin E2 (PGE2) signalling between IL‐1β⁺ macrophages and CAS cells, and among macrophages, via PTGES2/3–PTGER2 axes (Figure S5).

**FIGURE 4:**
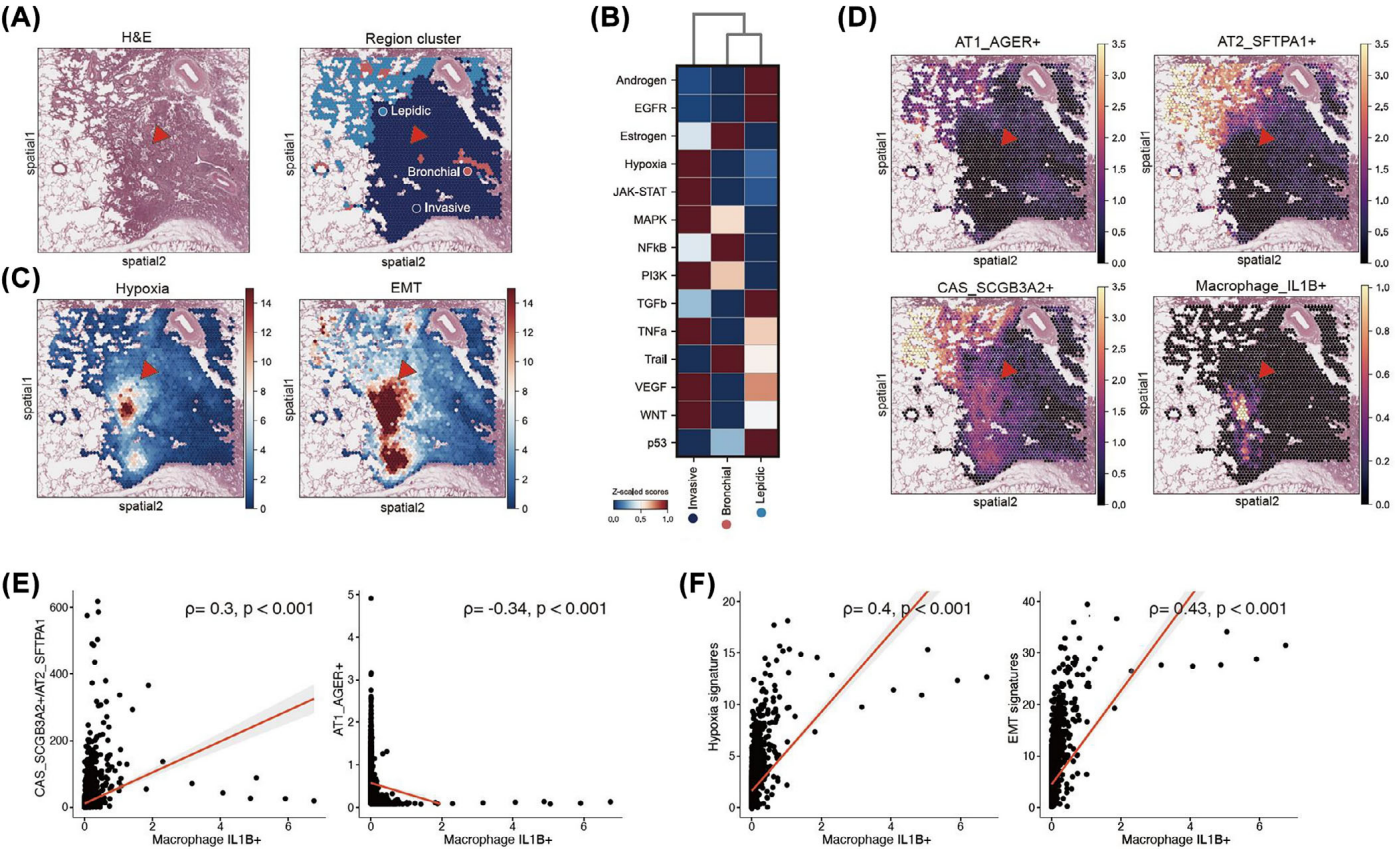
Spatial mapping of pathways and cell types from single‐cell RNA sequencing (scRNA‐seq) data. (A) Haematoxylin and eosin (H&E)‐stained image alongside results from unsupervised clustering of tissue analysed using Visium (SMC‐19). (B) Heatmap displaying the activity of 14 pathways, as inferred by PROGENy, for each cluster. The colour indicates the normalised scores (*Z*‐scaled) row‐wise. (C) Gene set signature scores for hypoxia and epithelial‒mesenchymal transition (EMT) in Visium slides. (D) Spatial gene expression plots showing the localisation and expression levels of alveolar type 1 (AT1) cells, alveolar type 2 (AT2) cells, cancer‐associated secretory (CAS) cells and interleukin 1 beta (IL‐1β^+^) macrophages. IL‐1β^+^ macrophages and CAS cells were co‐localised in specific invasive regions of the solid components, where EMT and hypoxia signatures were elevated (red arrow). (E) Correlation plot showing the association between the proportion of IL‐1β^+^ macrophages and CAS‐specific scores or AT1 cells. (F) Correlation plot showing the association between the proportion of IL‐1β^+^ macrophages and the pathway signature scores of hypoxia and EMT. The correlation coefficients (*ρ*) and *p*‐values are indicated in the plots.

Given the established role of IL‐1β in driving EMT and inflammation,^4^ our findings underscore its contribution to LUAD progression and suggest that IL‐1β‐targeted interventions may hold promise for early prevention.^5^, ^6^ Canakinumab, an IL‐1β antibody, reduced lung cancer risk and mortality in a trial originally targeting atherosclerosis.^5^ CAS cells, predominantly found in tumour regions, expressed SPINK1, CEACAM6 and CEACAM5—genes implicated in tumour proliferation and migration in lung cancer.^7^, ^8^, ^9^ Therapeutic antibodies targeting CEACAM6 and CEACAM5 are under clinical investigation for solid tumours, including CEACAM5‐positive lung cancer.^10^ While shown in EGFR‐mutant LUADs in never‐smokers, CAS and IL‐1β⁺ macrophage relevance in broader LUADs remains unclear.

In conclusion, CAS cells and IL‐1β^+^ macrophages play pivotal roles in the progression from preinvasive to invasive LUADs, positioning them as promising targets for cancer prevention and reversion strategies.

## AUTHOR CONTRIBUTIONS

*Conceptualisation*: Hong Kwan Kim, Woong‐Yang Park and Sang‐Won Um. *Resources*: Junsu Choe, Jinyeong Lim, Noeul Kang, Hong Kwan Kim and Sang‐Won Um. *Data curation*: Junsu Choe, Noeul Kang, Mina Hwang and Hong Kwan Kim. *Software*: Jinyeong Lim and Eun Seop Seo. *Formal analysis*: Junsu Choe, Jinyeong Lim, Noeul Kang, Eun Seop Seo, Kyung Yeon Han, Woong‐Yang Park and Sang‐Won Um. *Supervision*: Hong Kwan Kim, Woong‐Yang Park and Sang‐Won Um. *Funding acquisition*: Sang‐Won Um. *Validation*: Mina Hwang, Kyung Yeon Han, Se‐Hoon Lee, Myung‐Ju Ahn, Yoon‐La Choi, Hong Kwan Kim, Woong‐Yang Park and Sang‐Won Um. *Investigation*: Junsu Choe, Jinyeong Lim, Noeul Kang and Sang‐Won Um. *Visualisation*: Junsu Choe, Jinyeong Lim, Noeul Kang and Eun Seop Seo. *Methodology*: Hong Kwan Kim, Woong‐Yang Park and Sang‐Won Um. *Writing—original draft*: Junsu Choe, Jinyeong Lim, Noeul Kang, Eun Seop Seo and Sang‐Won Um. *Project administration*: Hong Kwan Kim, Woong‐Yang Park and Sang‐Won Um. *Writing—review and editing*: Junsu Choe, Jinyeong Lim, Noeul Kang, Eun Seop Seo, Mina Hwang, Kyung Yeon Han, Se‐Hoon Lee, Myung‐Ju Ahn, Yoon‐La Choi, Hong Kwan Kim, Woong‐Yang Park and Sang‐Won Um.

## CONFLICT OF INTEREST STATEMENT

The authors declare they have no conflicts of interest.

## FUNDING INFORMATION

This work was supported by the National Research Foundation of Korea grant funded by the Korean Government (MSIT) (RS‐2025‐00557023).

## ETHICS STATEMENT

Written informed consent was acquired from all participants under institutional review board (IRB)‐approved protocols at Samsung Medical Center (IRB no. 2017‐10‐022).

## Supporting information



Supporting Information

## Data Availability

The data generated in this study are included in the article and its Supporting Information, which contain raw and processed single‐cell RNA sequencing data, WES data and whole WTS data. All datasets have been deposited in the Gene Expression Omnibus (accession no. GSE288479). The codes used for data processing and analysis are available from the corresponding author upon request.
