# Supplementary material for: Novel cancer‐associated secretory cells and IL‐1β+ macrophages as key players in early lung adenocarcinoma progression in female never‐smokers
Source: Clin Transl Med. 2025 Aug 18;15(8):e70433. doi: 10.1002/ctm2.70433 (PMC12360329; doi:10.1002/ctm2.70433)
Supplement: Supplementary file 1 — Supporting Information [file CTM2-15-e70433-s001.docx]

**
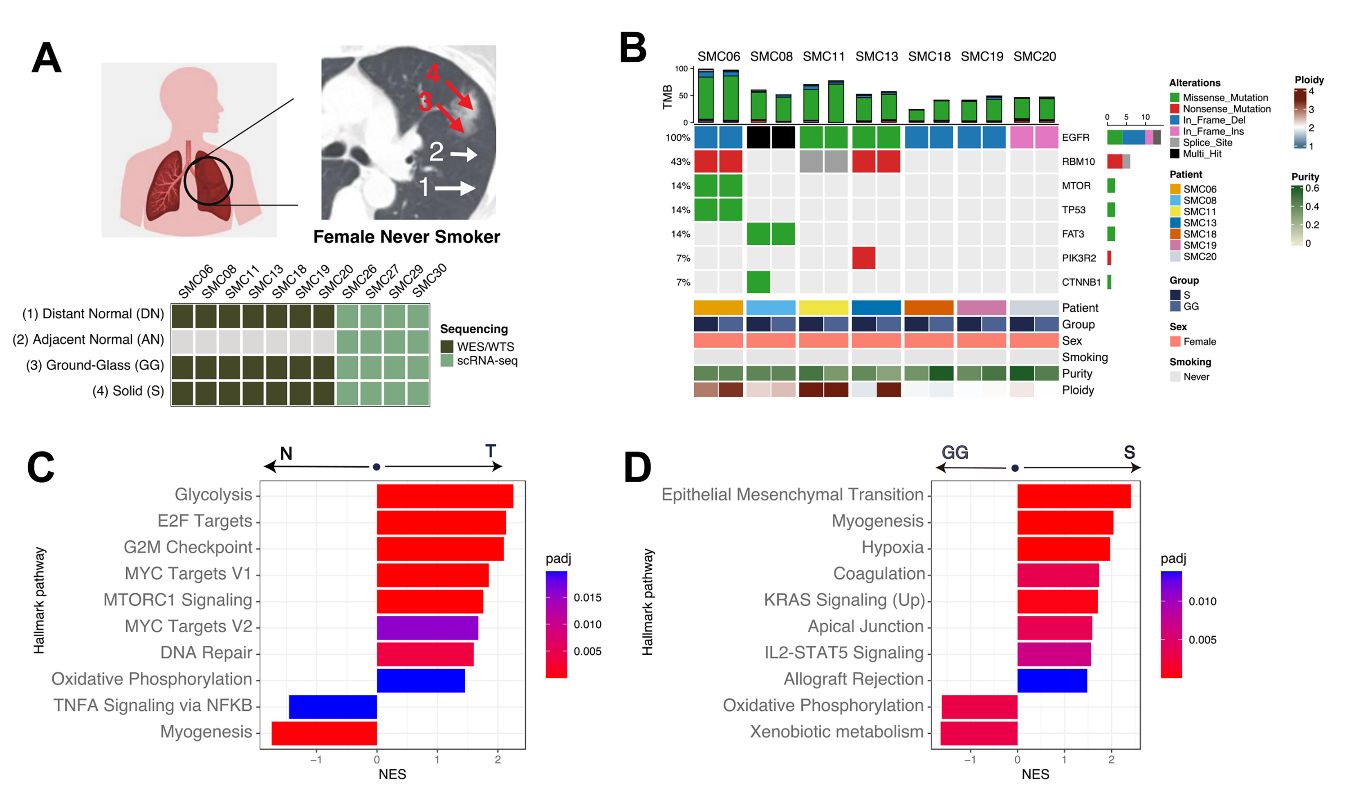
**

**Figure S1. Genomic and transcriptomic characteristics of different components in part-solid-type lung adenocarcinoma**

(A) Samples from different components of lung adenocarcinoma with part-solid lesions: DN, AN, GG, and S. WES/WTS or scRNA-seq was applied to each sample. (B) Oncoplot showing S and GG from WES. For each study subject, the left and right panels represent S and GG, respectively. (C) GSEA with transcriptomic data showing enriched gene sets in tumors (S and GG) compared to matched normal regions. (D) GSEA results indicating that the S component exhibited several enriched hallmark gene sets, such as EMT-related and hypoxia pathways, compared to GG. The horizontal axis represents positive (red) and negative (blue) normalized enrichment scores (NES).

AN, adjacent normal lung tissue; DN, distant normal lung tissue; EMT, epithelial-mesenchymal transition; GG, ground glass component of tumor; GSEA, gene set enrichment analysis; scRNA-seq, single-cell RNA sequencing; S, solid component of tumor; WES, whole-exome sequencing; WTS, whole-transcriptome sequencing.

**
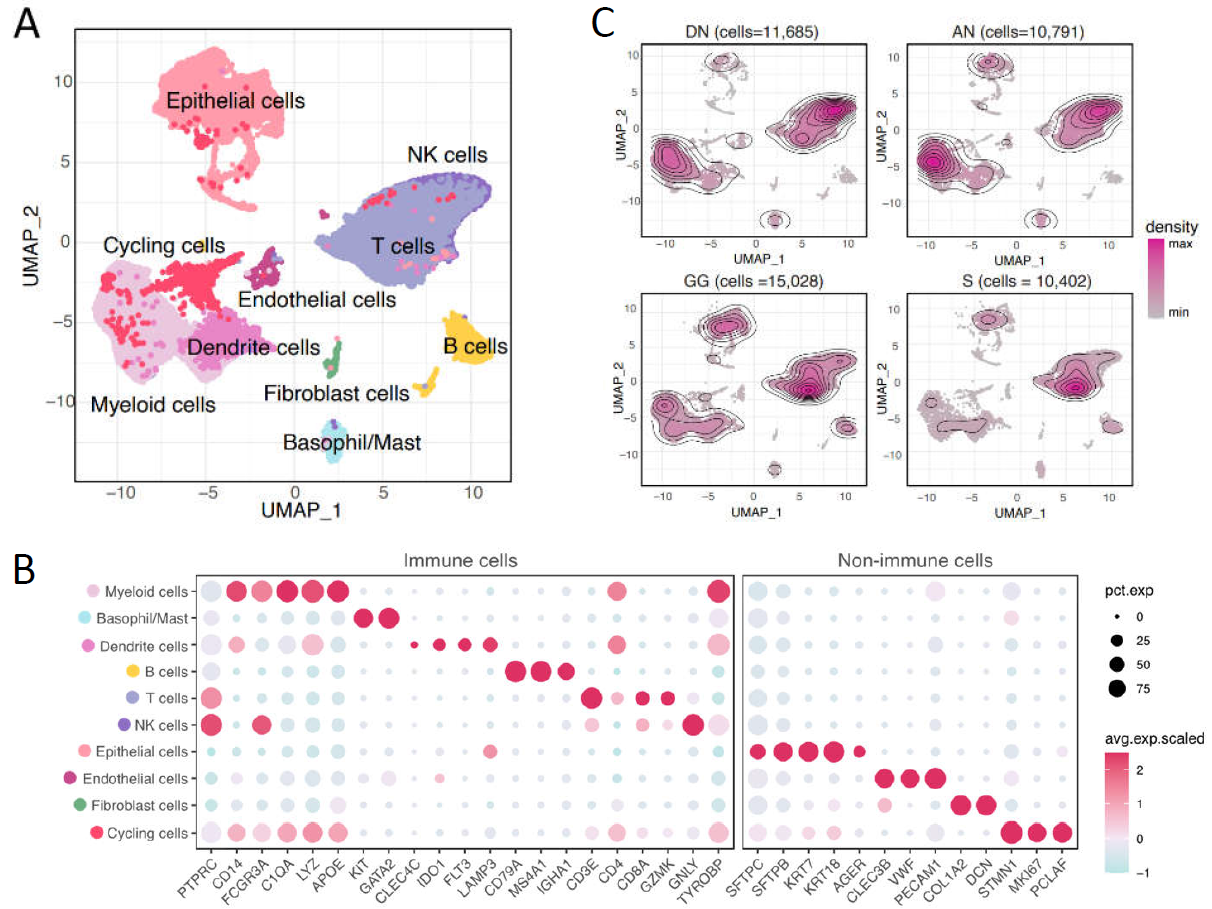
** **
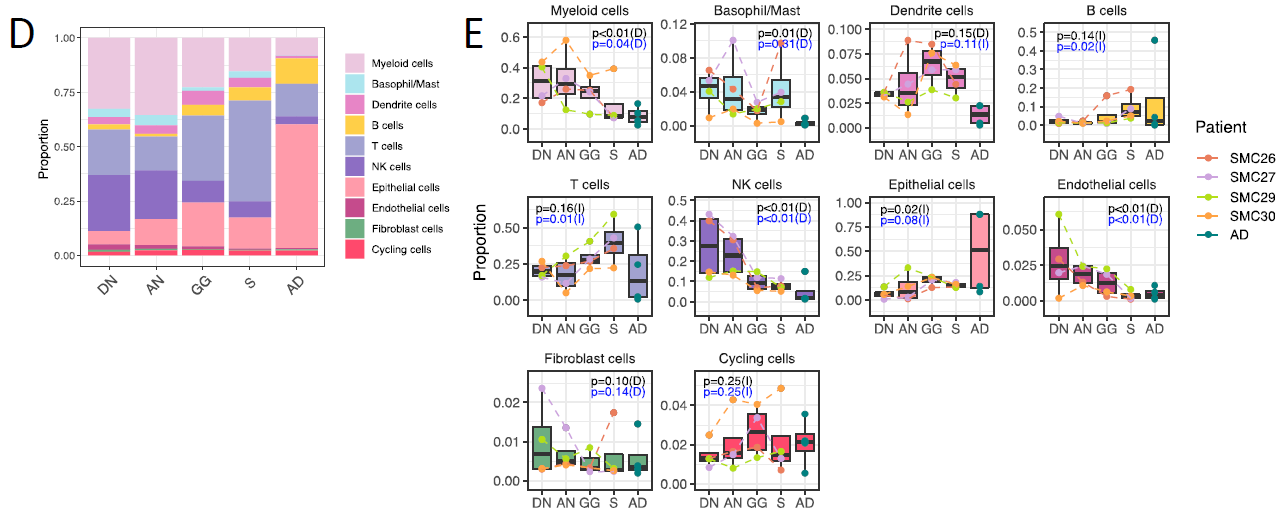
**

**Figure S2.** **Overview of single-cell RNA sequencing analysis.**

(A) UMAP plot showing 46,792 single cells from four components of part-solid-type adenocarcinoma and AD, colored according to the 10 major cell types. Each dot represents a single cell, colored based on cell type. (B) Dot plot of marker gene expression for the 10 major cell types. The dot size is proportional to the fraction of cells expressing each gene, and the color intensity represents the relative expression levels of genes. (C) Cell density plot displaying the cell distribution across the four components of part-solid-type adenocarcinoma (D) Bar plots showing the average percentages of each major cell subtype across DN, AN, GG, S, and AD. (E) Proportions of major cells in part-solid-type adenocarcinomas (n = 4) and ADs (n=3). The color of points and lines indicates patient-specific data. Jonckheere–Terpstra tests were conducted across components, with (D) representing a decreasing trend and (I) representing an increasing trend. Trends across the four components of part-solid-type adenocarcinomas are displayed in black, whereas trends including the four components of part-solid-type adenocarcinomas and ADs are shown in blue. AD, advanced lung adenocarcinoma; AN, adjacent normal lung; DN, distant normal lung; GG, ground glass component of tumor; S, solid component of tumor; UMAP, uniform manifold approximation and projection.

**
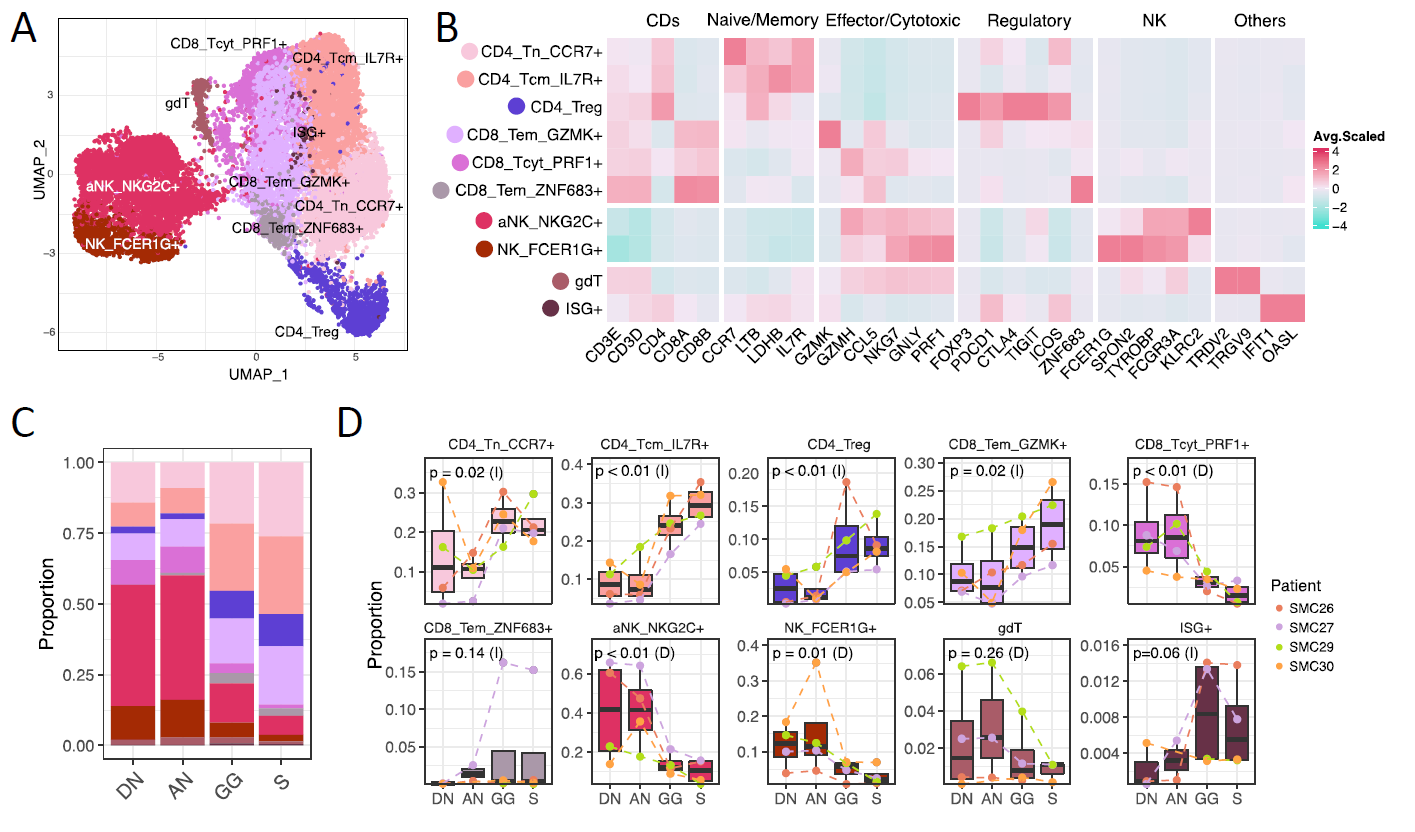
**

**Figure S3. Composition and characterization of T and NK subsets in four components of part-solid-type LUAD**

(A) UMAP plot of 22,943 T and NK cells revealing 10 subtypes. Each dot represents a single cell and is colored according to cell type. (B) Dot plot showing the expression of marker genes for the 10 T and NK subset cell types. The dot size is proportional to the fraction of cells expressing each specific gene, while the color intensity represents the relative expression of each gene. (C) Bar plots showing the proportion of each T and NK cell subtype across DN, AN, GG, and S. Cell subtypes are represented in different colors. (D) Boxplots illustrating the trends of T and NK subset cell types from DN to S. The color of points and lines indicates patient-specific data (n=4). Jonckheere–Terpstra tests were performed across the four components, with (D) indicating a decreasing trend and (I) indicating an increasing trend.

AN, adjacent normal lung; DN, distant normal lung; GG, ground glass component of tumor; LUAD, lung adenocarcinoma; S, solid component of tumor; UMAP, uniform manifold approximation and projection.


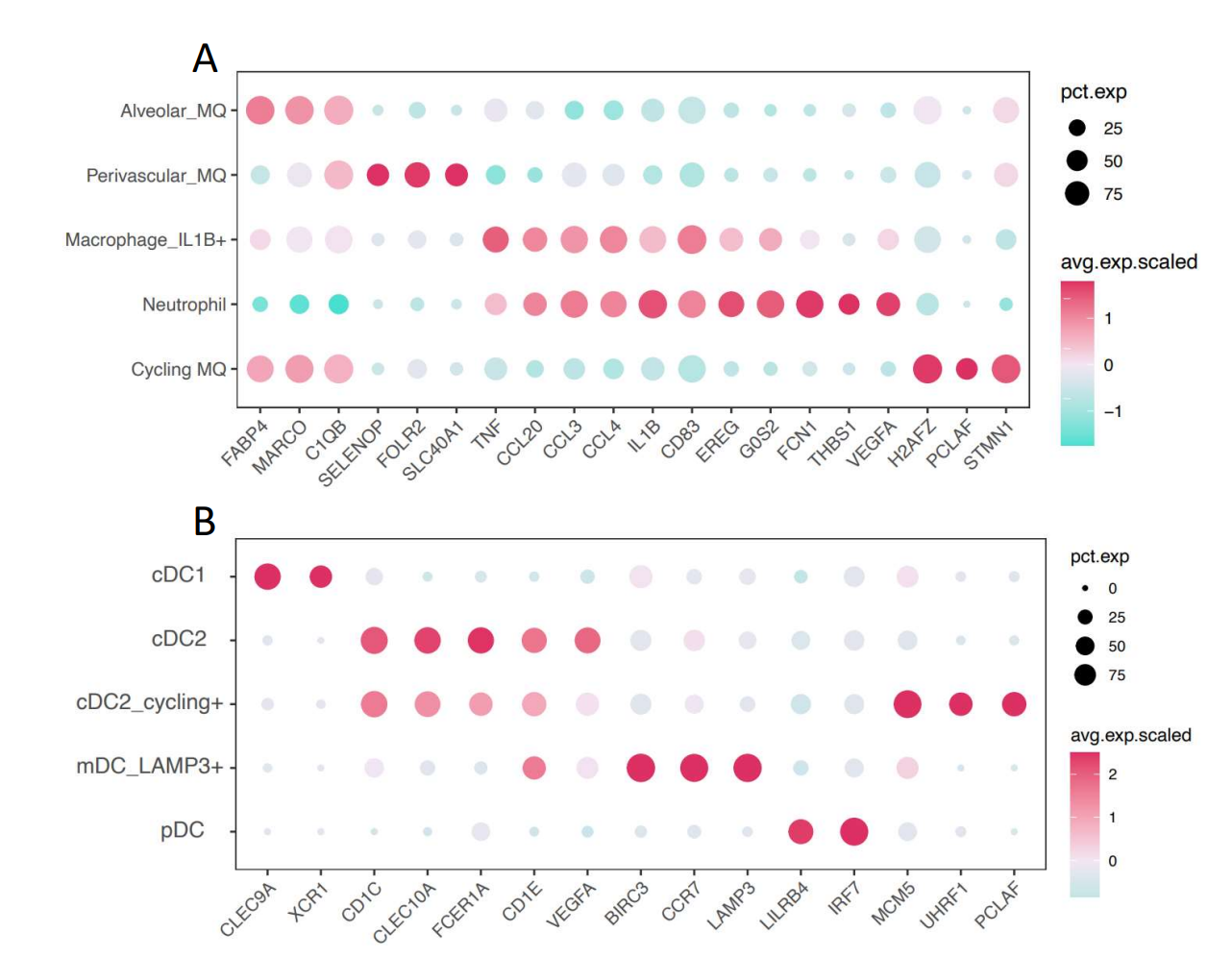


Figure S4. Canonical markers used to annotate myeloid cell subtypes. (A) Dot plot of gene marker expression among macrophages and neutrophils and (B) by dendritic cell subtype.


Figure S5. Prostaglandin E2 signaling between IL-1β⁺ macrophages and CAS_SCGB3A2+ cells. Dot plot showing PTGES2–PTGER2 and PTGES3–PTGER2 interactions between IL-1β⁺ macrophages and CAS_SCGB3A2⁺ cells in tumor (SS, G) and normal (DN, AN) regions. Dot color and size indicates scaled expression; dot outline color indicates significance.

**Table S1**. Clinicopathological characteristics of patients (n = 11)

| **Characteristics** | **Total (n = 11)** | **WES & WTS (n = 7)** | **scRNA-seq (n = 4)** |
| --- | --- | --- | --- |
| Study ID |  | SMC-6, 8, 11, 13, 18, 19, 20 | SMC-26, 27, 29, 30 |
| Age at diagnosis, years | 64 (49–79) | 63 (49–79) | 66 (55–68) |
| Tumor size, mm | 32 (22–38) | 32 (23 – 38) | 28 (22–35) |
| Solid portion size, mm | 21 (6–32) | 26 (17–32) | 13.5 (6–29) |
| Tumor location |  |  |  |
| Left upper lobe | 4 (36) | 4 (57) | 0 |
| Right upper lobe | 3 (27) | 0 | 3(7) |
| Right middle lobe | 1 (9) | 1 (14) | 0 |
| Right lower lobe | 3 (27) | 2 (29) | 1 (25) |
| TNM stage^*^ |  |  |  |
| IA2 | 1 (9) | 1 (14) | 0 |
| IA3 | 7 (64) | 4 (57) | 3 (75) |
| IB | 3 (27) | 2 (29) | 1 (25) |
| Histological patterns |  |  |  |
| Acinar | 1 (9) | 0 | 1 (25) |
| Acinar and lepidic | 7 (64) | 5 (71) | 2 (50) |
| Acinar and papillary | 2 (18) | 2 (29) | 0 |
| Acinar, lepidic, and papillary | 1 (9) | 0 | 1 (25) |
| *EGFR* mutation type^†^ |  |  |  |
| Deletion mutation in exon 19 | 4 (36) | 3 (43) | 1 (25) |
| Missense mutation in exon 21 | 5 (45) | 3 (43) | 2 (50) |
| Uncommon mutation^‡^ | 1 (9) | 1 (14) | 0 |
| *Eighth edition of the American Joint Commission on Cancer TNM staging system for non-small cell lung cancer.  †One patient from the scRNA group did not harbor an *EGFR* mutation.  ^‡^*p.P772_H773 dup* mutation was detected by WES.  *EGFR*, epidermal growth factor receptor; WES, whole-exome sequencing; scRNA-seq, single-cell RNA sequencing. | | | |

Table S2. Clinical characteristics of each patient (n = 11)

| Patient ID | SMC06 | SMC08 | SMC11 | SMC13 | SMC18 | SMC19 | SMC20 | SMC26 | SMC27 | SMC29 | SMC30 |
| --- | --- | --- | --- | --- | --- | --- | --- | --- | --- | --- | --- |
| Age, years | 57 | 64 | 70 | 79 | 49 | 66 | 56 | 55 | 67 | 68 | 64 |
| Location | LUL | LUL | LUL | RML | LUL | RLL | RLL | RUL | RUL | RUL | RLL |
| Total size, mm | 36 | 32 | 37 | 24 | 25 | 28 | 28 | 32 | 62 | 22 | 25 |
| Solid component size, mm | 21 | 18 | 29 | 27 | 26 | 17 | 32 | 11 | 29 | 16 | 6 |
| Pathological Stage | pT1cN0M0 | pT1cN0M0 | pT2N0M0 | pT1cN0M0 | pT1bN0M0 | pT1cN0M0 | pT2aN0M0 | pT2aN0M0 | pT1cN0M0 | pT1cN0M0 | pT1cN0M0 |
| Histologic pattern | Acinar & papillary | Acinar & lepidic | Acinar & papillary | Acinar & lepidic | Acinar & lepidic | Acinar & lepidic | Acinar & lepidic | Acinar & lepidic | Acinar, lepidic & papillary | acinar | Acinar & lepidic |
| H&E staining for ground-glass region* | 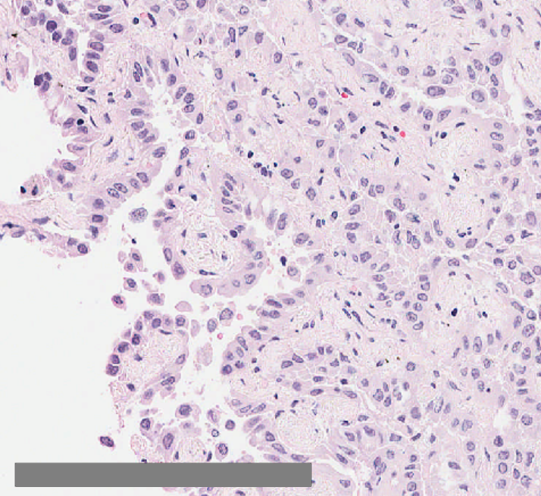 | 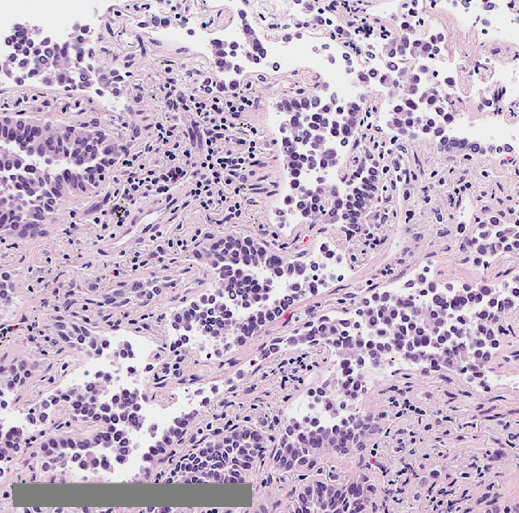 | 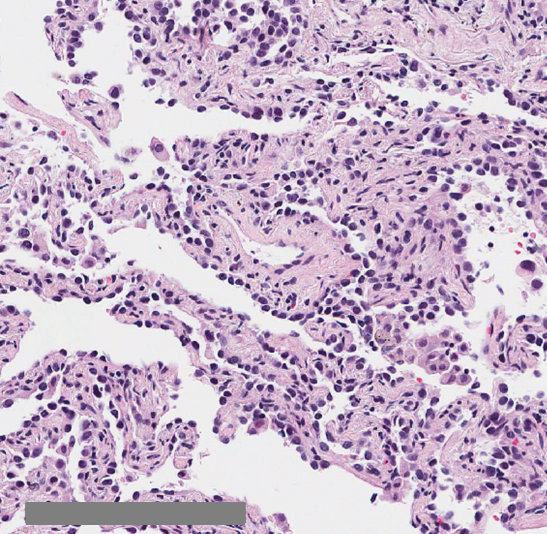 | 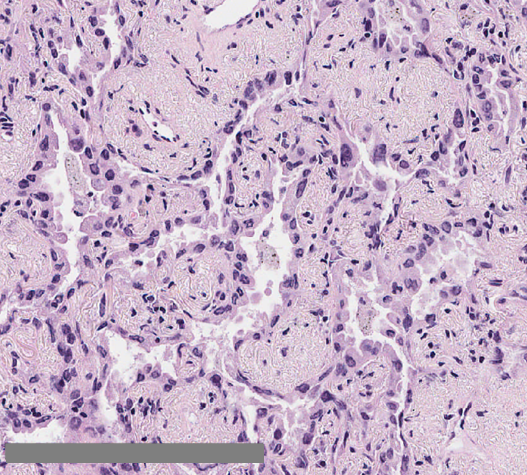 | 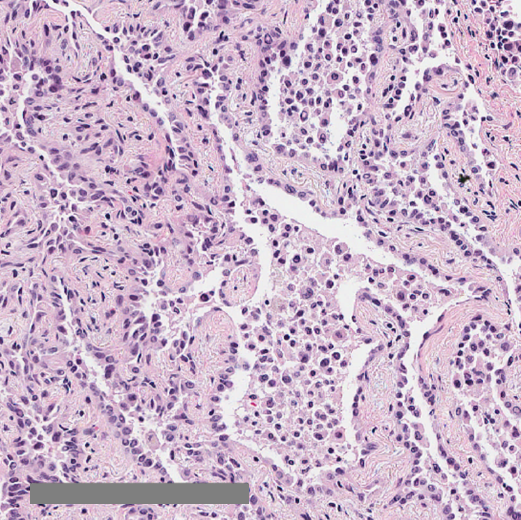 | 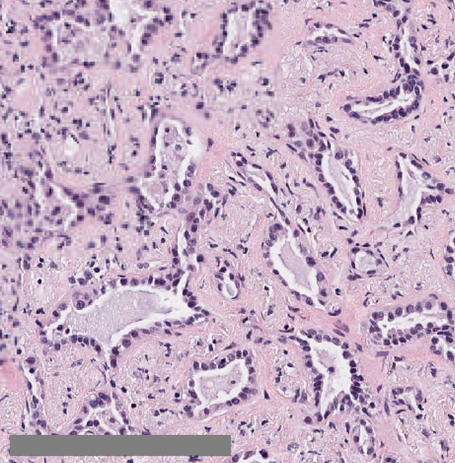 | 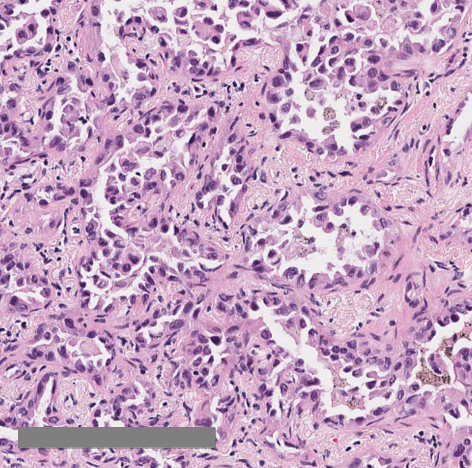 | 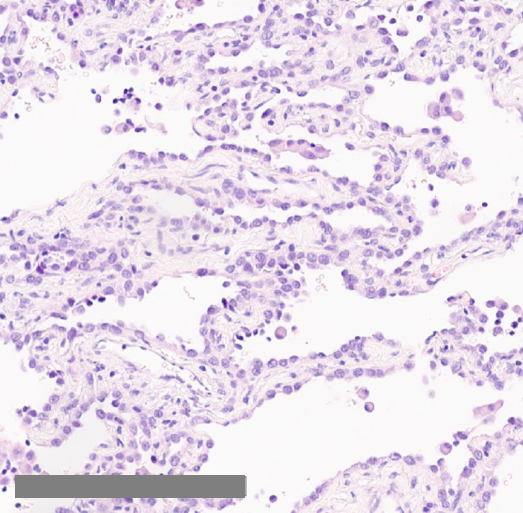 | 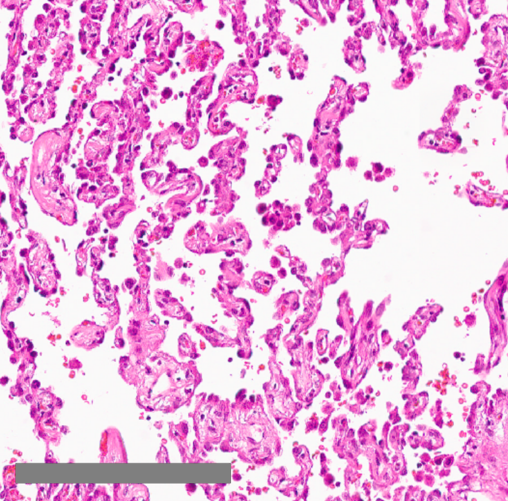 | 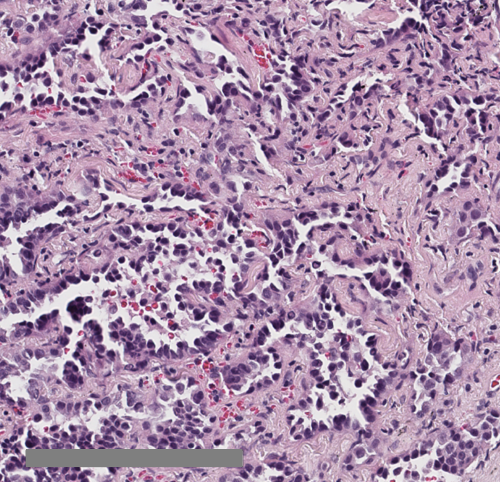 | 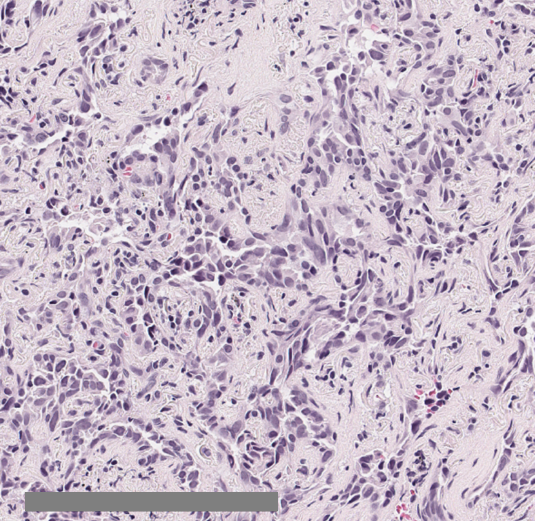 |
| H&E staining for solid region* | 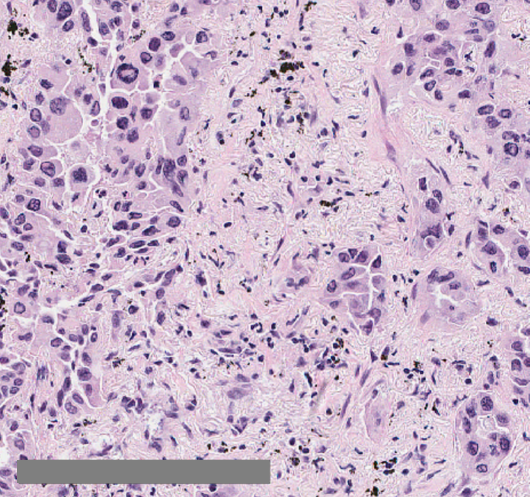 | 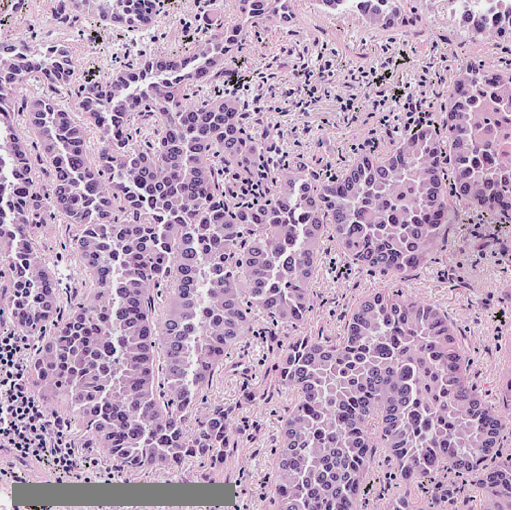 | 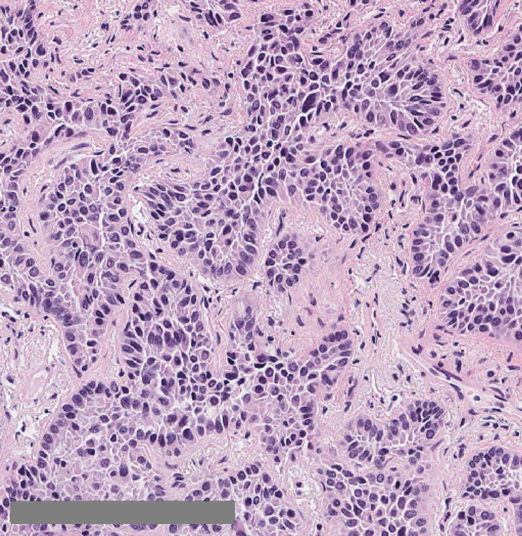 | 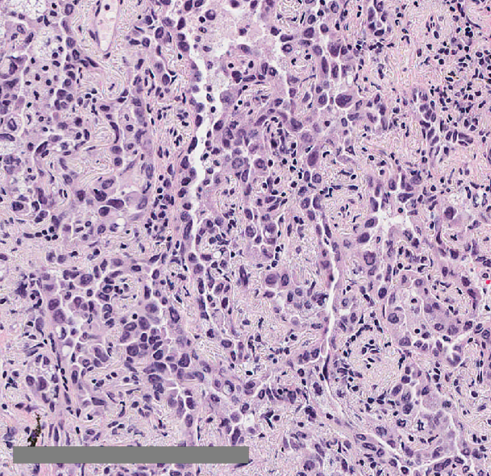 | 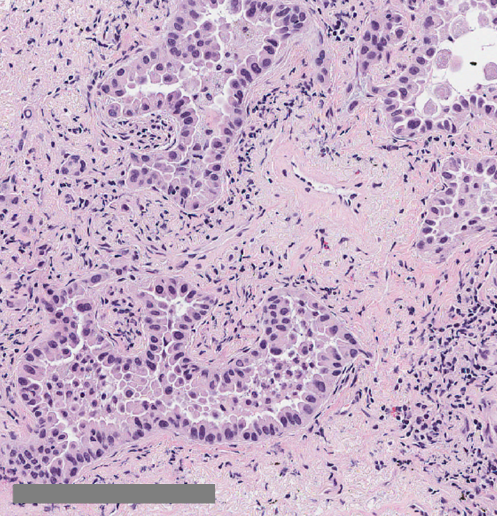 | 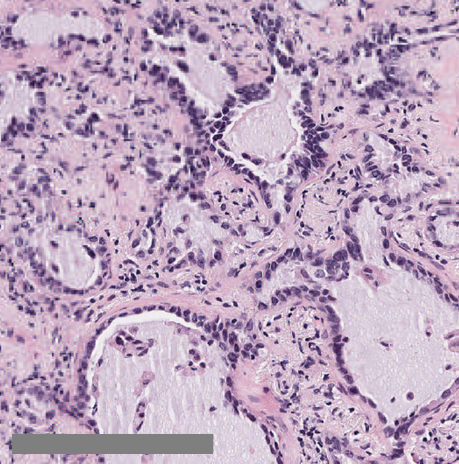 | 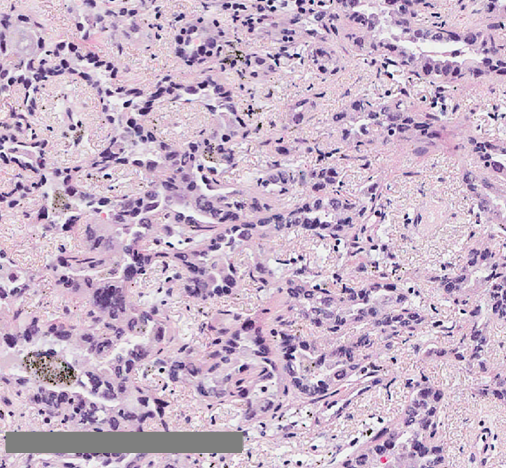 | 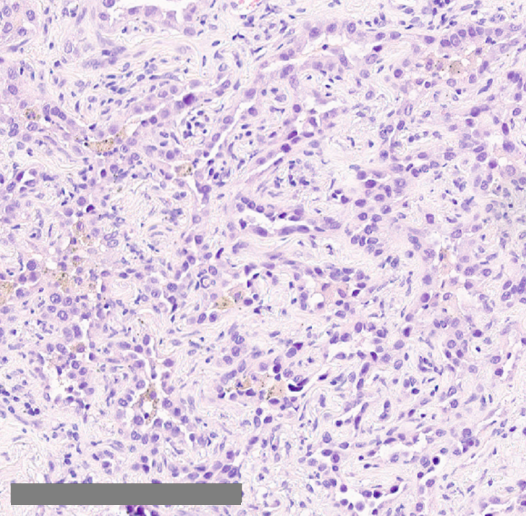 | 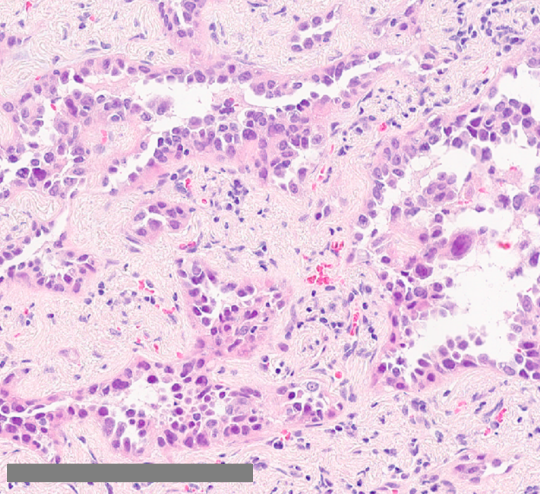 | 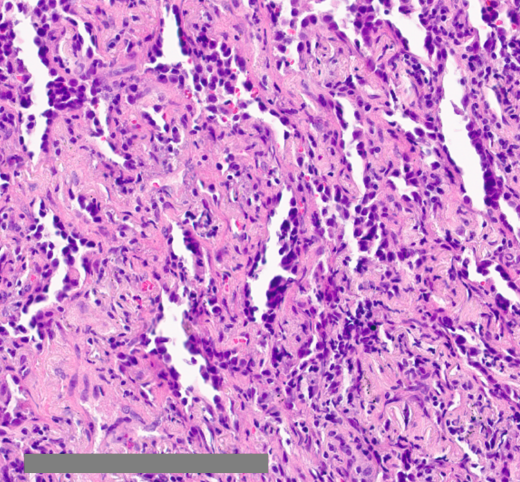 | 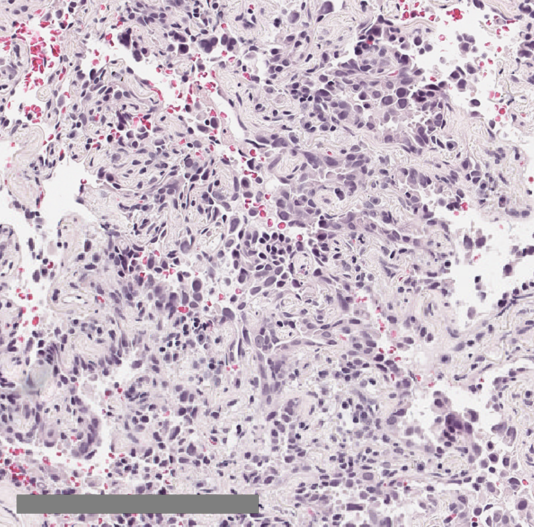 |
| EGFR mutation | Deletion mutation in exon 19 | Missense mutation in exon 21 (L858R) | Missense mutation in exon 21 (L858R) | Missense mutation in exon 21 (L858R) | Deletion mutation in exon 19 | Deletion mutation in exon 19 | p.P772_H773 dup | Missense mutation in exon 21 (L858R) | Missense mutation in exon 21 (L858R) | Deletion mutation in exon 19 | (-) |
| Chest CT finding | 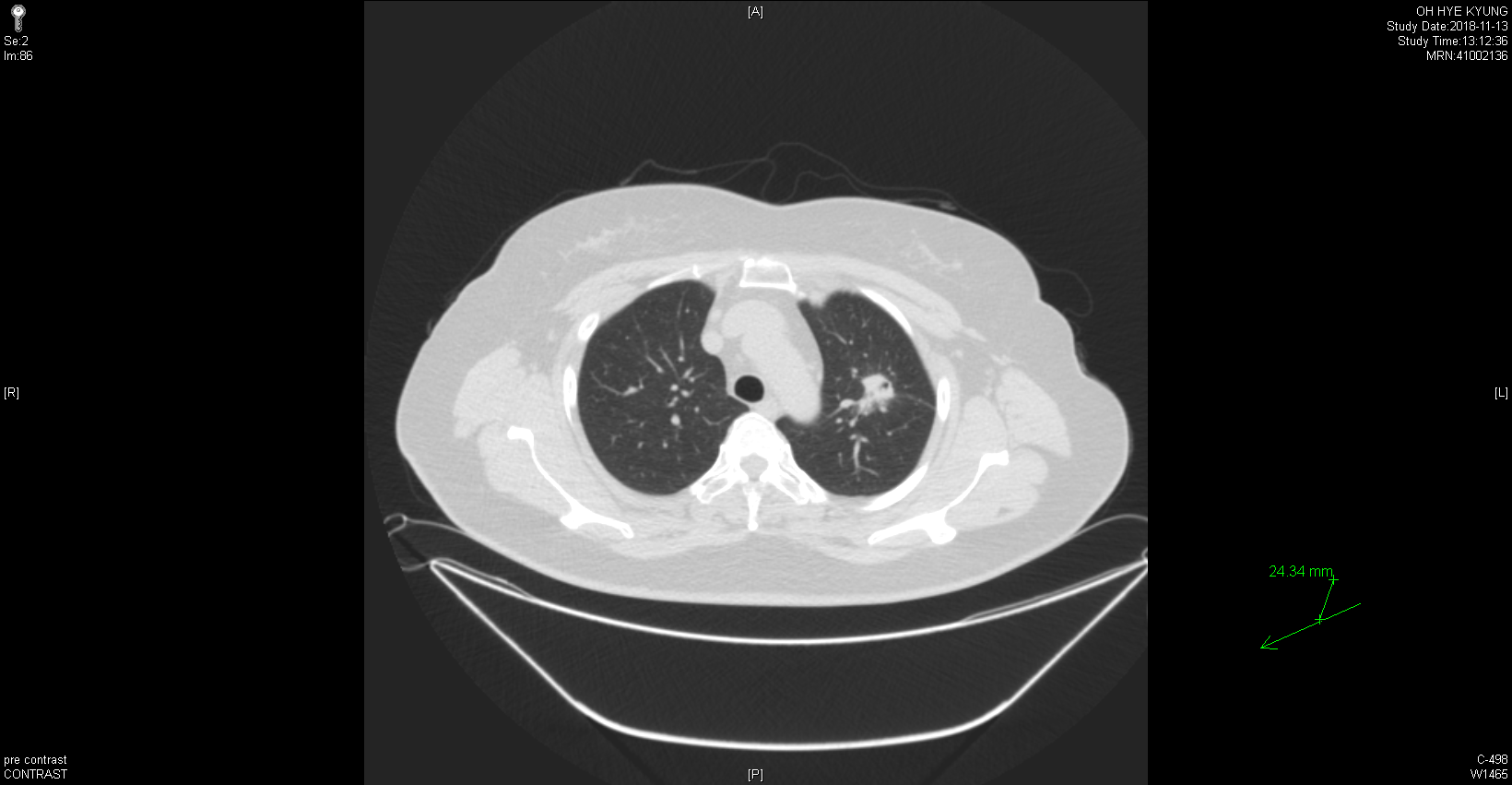 | 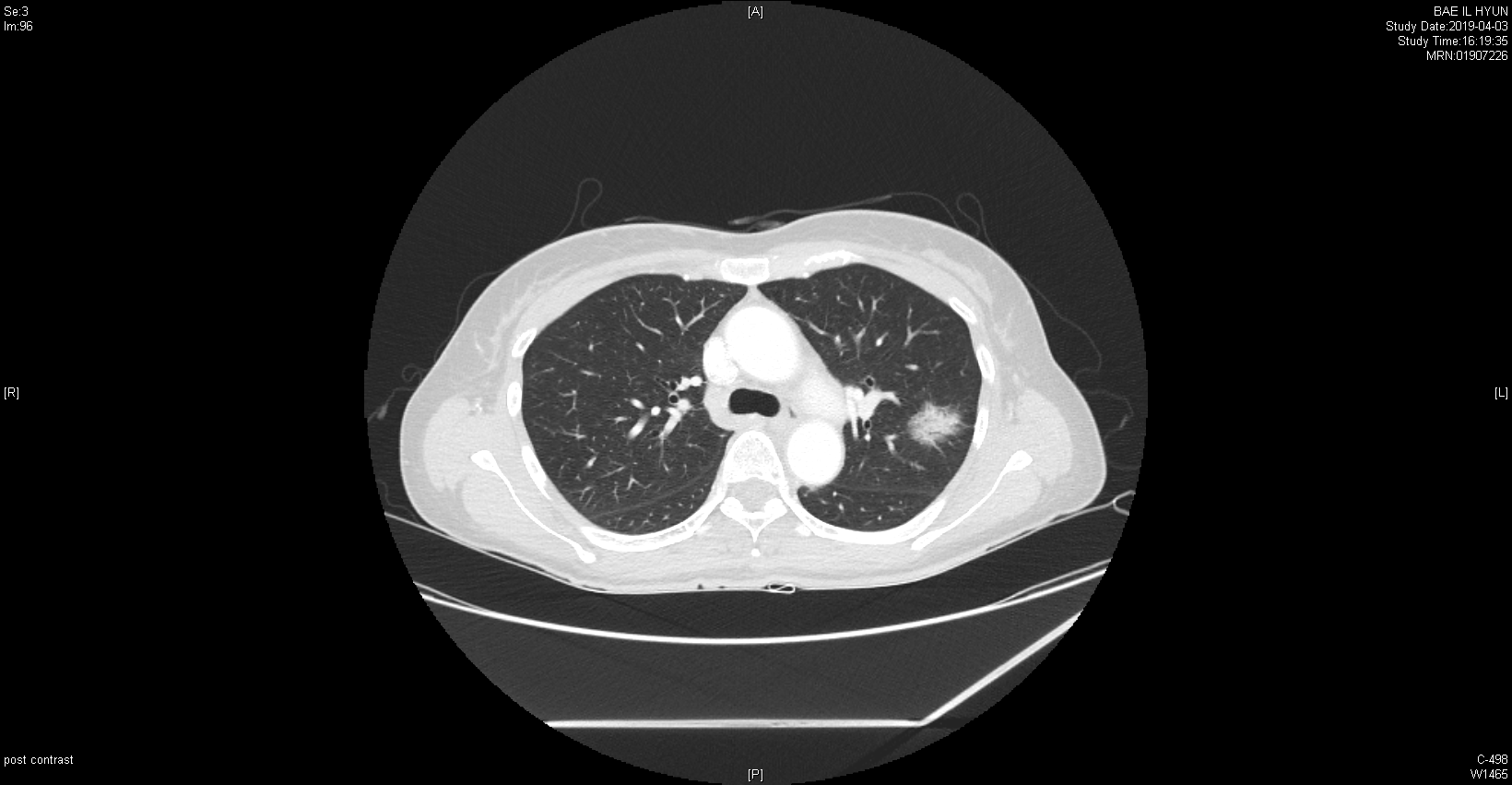 | 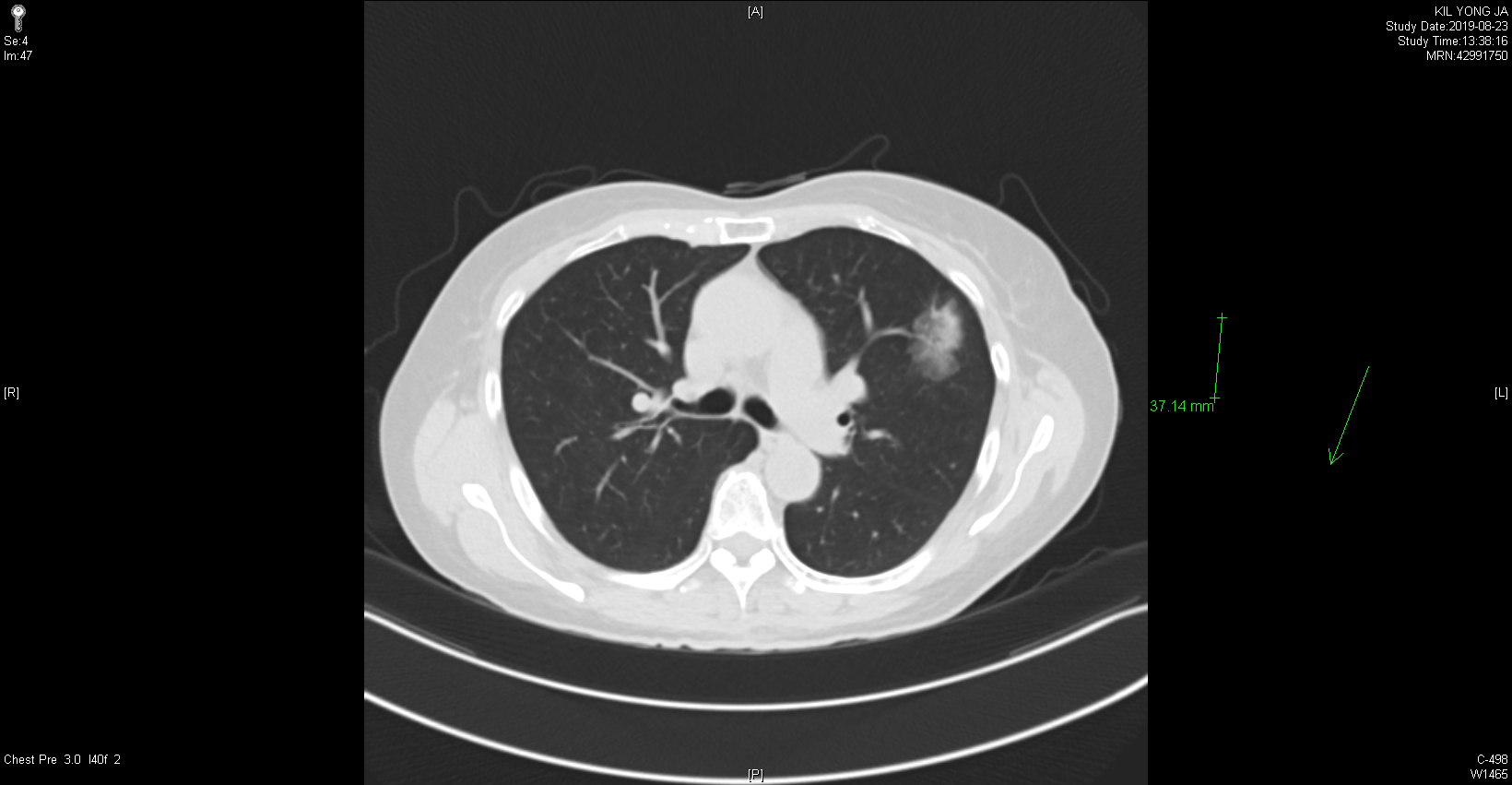 | 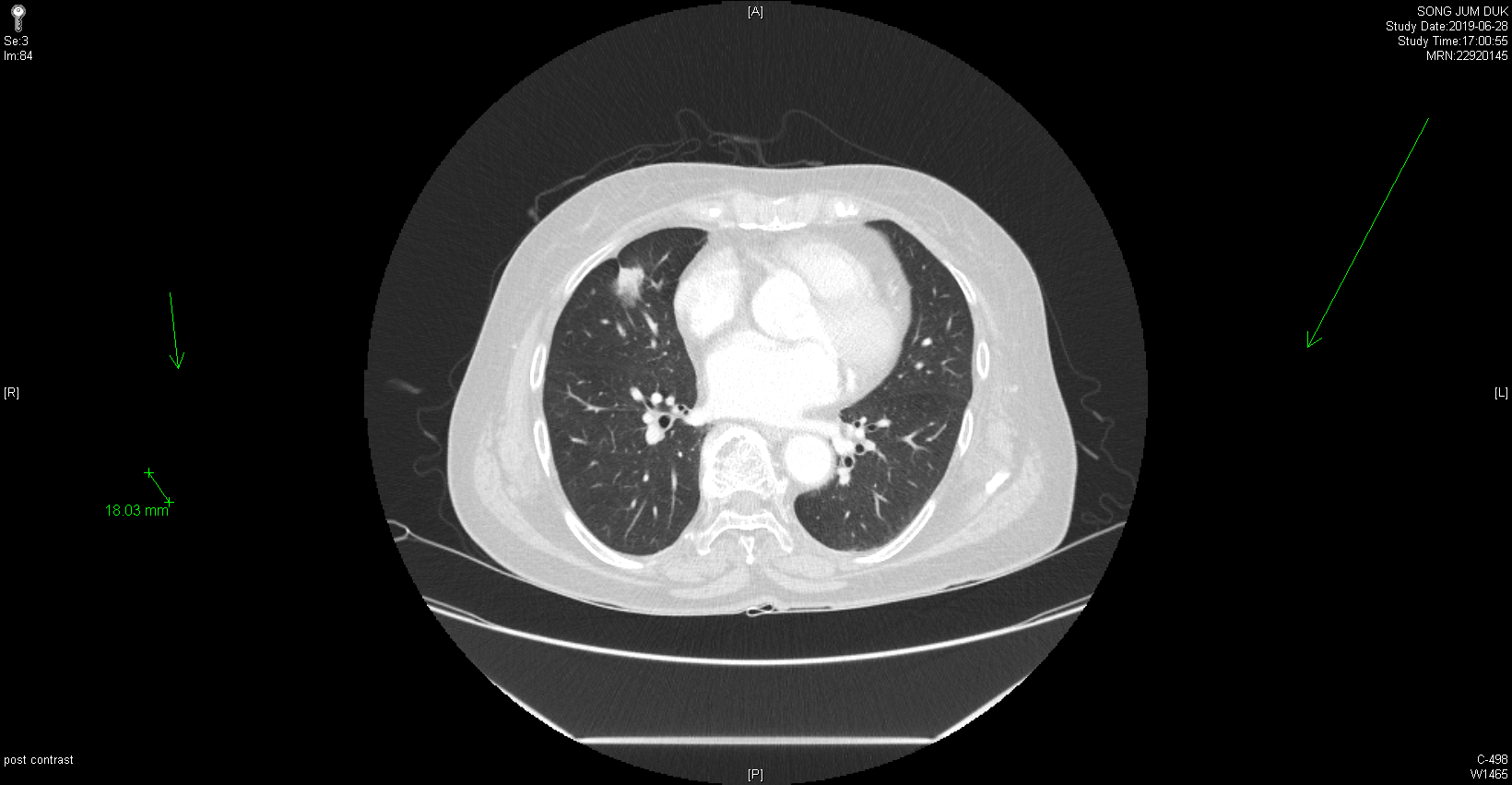 | 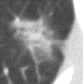 | 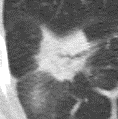 | 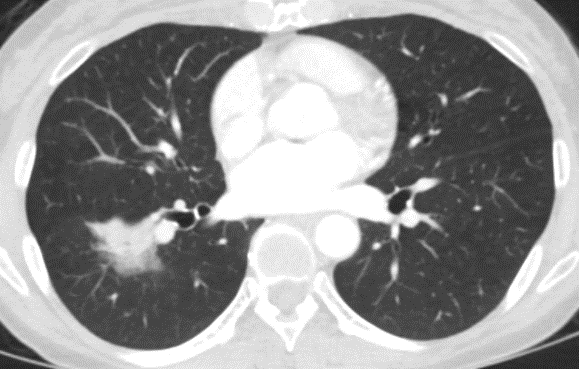 | 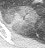 | 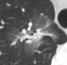 | 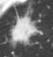 | 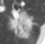 |

*Scale bars in the pathology figures represent 200 micrometers.

CT, computed tomography; EGFR, epidermal growth factor receptor; LUL, left upper lobe; RML, right middle lobe; RLL, right lower lobe; RUL, right upper lobe; H&E, hematoxylin and eosin.

Table S3. Differential expressed gene list between CAS cells and AT2 cells

| gene | p_val | avg_log2FC | pct.1 | pct.2 | p_val_adj | DEG |
| --- | --- | --- | --- | --- | --- | --- |
| ABCC3 | 0 | 1.31920962 | 0.688 | 0.049 | 0 | Up-DEG |
| APOD | 0 | 1.98504195 | 0.522 | 0.019 | 0 | Up-DEG |
| CEACAM5 | 0 | 2.63294643 | 0.588 | 0.015 | 0 | Up-DEG |
| CEACAM6 | 0 | 2.75289627 | 0.849 | 0.104 | 0 | Up-DEG |
| CRABP2 | 0 | 1.96905875 | 0.57 | 0.01 | 0 | Up-DEG |
| GDF15 | 0 | 2.91577727 | 0.88 | 0.219 | 0 | Up-DEG |
| KRT7 | 0 | 1.96528687 | 0.85 | 0.253 | 0 | Up-DEG |
| MDK | 0 | 2.19692228 | 0.736 | 0.093 | 0 | Up-DEG |
| S100A11 | 0 | 1.39968845 | 0.967 | 0.854 | 0 | Up-DEG |
| SOX4 | 0 | 1.79535605 | 0.809 | 0.218 | 0 | Up-DEG |
| TAGLN2 | 0 | 1.84646066 | 0.93 | 0.609 | 0 | Up-DEG |
| TMSB4X | 0 | 1.49977207 | 0.995 | 0.976 | 0 | Up-DEG |
| MUC21 | 1.59737308116383e-315 | 1.32305631 | 0.491 | 0.022 | 3.93225331390099e-311 | Up-DEG |
| ELOB | 8.69E-307 | 1.22247439 | 0.872 | 0.613 | 2.14E-302 | Up-DEG |
| WFDC2 | 5.65E-302 | 2.00920528 | 0.944 | 0.778 | 1.39E-297 | Up-DEG |
| S100A10 | 1.07E-301 | 1.52577395 | 0.94 | 0.782 | 2.63E-297 | Up-DEG |
| CD24 | 3.43E-295 | 1.66453373 | 0.702 | 0.18 | 8.45E-291 | Up-DEG |
| KRT18 | 6.54E-292 | 1.32688191 | 0.882 | 0.587 | 1.61E-287 | Up-DEG |
| CLIC6 | 1.15E-287 | 1.03804665 | 0.597 | 0.092 | 2.82E-283 | Up-DEG |
| TNFRSF12A | 5.93E-275 | 1.47126908 | 0.643 | 0.151 | 1.46E-270 | Up-DEG |
| PCSK1N | 2.23E-269 | 1.3090571 | 0.547 | 0.079 | 5.50E-265 | Up-DEG |
| OCIAD2 | 3.93E-269 | 1.3556981 | 0.73 | 0.277 | 9.66E-265 | Up-DEG |
| TM4SF1 | 1.06E-268 | 1.59153374 | 0.758 | 0.305 | 2.60E-264 | Up-DEG |
| TXN | 8.62E-268 | 1.38862004 | 0.881 | 0.672 | 2.12E-263 | Up-DEG |
| CRIP2 | 3.88E-259 | 1.23501071 | 0.579 | 0.102 | 9.55E-255 | Up-DEG |
| SMIM22 | 1.49E-244 | 1.23020995 | 0.77 | 0.369 | 3.67E-240 | Up-DEG |
| APP | 3.60E-244 | 1.2790568 | 0.807 | 0.416 | 8.85E-240 | Up-DEG |
| PPDPF | 1.22E-235 | 1.47922667 | 0.94 | 0.805 | 3.01E-231 | Up-DEG |
| MXRA8 | 2.32E-228 | 1.01178349 | 0.359 | 0.011 | 5.71E-224 | Up-DEG |
| ACTB | 5.99E-227 | 1.05389636 | 0.988 | 0.95 | 1.48E-222 | Up-DEG |
| IGFBP2 | 3.03E-226 | 1.30058739 | 0.442 | 0.044 | 7.46E-222 | Up-DEG |
| KRT8 | 1.36E-222 | 1.17031833 | 0.861 | 0.617 | 3.34E-218 | Up-DEG |
| LY6E | 2.72E-222 | 1.59713281 | 0.786 | 0.382 | 6.70E-218 | Up-DEG |
| S100A6 | 1.31E-218 | 1.62677433 | 0.973 | 0.908 | 3.22E-214 | Up-DEG |
| DSTN | 1.94E-212 | 1.0130271 | 0.911 | 0.743 | 4.77E-208 | Up-DEG |
| TMSB10 | 1.19E-211 | 1.18236107 | 0.969 | 0.893 | 2.93E-207 | Up-DEG |
| PPP1R14B | 7.15E-210 | 1.03087035 | 0.64 | 0.201 | 1.76E-205 | Up-DEG |
| NAP1L1 | 1.93E-200 | 1.0469539 | 0.751 | 0.35 | 4.75E-196 | Up-DEG |
| SELENOW | 3.11E-196 | 1.03336858 | 0.912 | 0.747 | 7.65E-192 | Up-DEG |
| SPINK1 | 9.69E-196 | 3.59198609 | 0.338 | 0.017 | 2.39E-191 | Up-DEG |
| SUB1 | 3.20E-191 | 1.01021427 | 0.792 | 0.48 | 7.87E-187 | Up-DEG |
| SOD1 | 1.53E-188 | 1.04708667 | 0.784 | 0.518 | 3.76E-184 | Up-DEG |
| SPINT2 | 1.16E-185 | 1.09954273 | 0.879 | 0.71 | 2.85E-181 | Up-DEG |
| TIMP1 | 3.21E-176 | 1.11502334 | 0.531 | 0.134 | 7.90E-172 | Up-DEG |
| TFF3 | 3.10E-172 | 1.19305794 | 0.376 | 0.047 | 7.64E-168 | Up-DEG |
| RAB11FIP1 | 1.13E-163 | 1.07421183 | 0.708 | 0.336 | 2.78E-159 | Up-DEG |
| CXCL14 | 3.15E-162 | 1.9948395 | 0.287 | 0.014 | 7.75E-158 | Up-DEG |
| TXNDC17 | 4.20E-156 | 1.06044925 | 0.763 | 0.463 | 1.03E-151 | Up-DEG |
| AQP3 | 2.65E-154 | 1.31481027 | 0.854 | 0.61 | 6.52E-150 | Up-DEG |
| IFI6 | 3.79E-154 | 1.43361062 | 0.585 | 0.201 | 9.34E-150 | Up-DEG |
| CLDN3 | 4.52E-154 | 1.07009355 | 0.665 | 0.317 | 1.11E-149 | Up-DEG |
| ARHGDIB | 5.36E-154 | 1.27362801 | 0.635 | 0.297 | 1.32E-149 | Up-DEG |
| COL1A1 | 5.68E-153 | 1.17457939 | 0.371 | 0.058 | 1.40E-148 | Up-DEG |
| LAPTM4B | 2.37E-149 | 1.02086204 | 0.689 | 0.385 | 5.83E-145 | Up-DEG |
| CRIP1 | 1.99E-147 | 1.31414188 | 0.74 | 0.411 | 4.89E-143 | Up-DEG |
| TIMP3 | 9.10E-147 | 1.00228431 | 0.292 | 0.023 | 2.24E-142 | Up-DEG |
| IFI27 | 2.01E-141 | 1.92472359 | 0.613 | 0.266 | 4.95E-137 | Up-DEG |
| TRPM4 | 2.51E-136 | 1.13998026 | 0.365 | 0.069 | 6.18E-132 | Up-DEG |
| TRAM1 | 4.32E-130 | 1.25514685 | 0.756 | 0.504 | 1.06E-125 | Up-DEG |
| KRT19 | 7.82E-125 | 1.08764586 | 0.826 | 0.61 | 1.92E-120 | Up-DEG |
| RNASE1 | 1.39E-119 | 1.09736704 | 0.96 | 0.843 | 3.42E-115 | Up-DEG |
| LGALS1 | 4.13E-119 | 1.09006553 | 0.453 | 0.139 | 1.02E-114 | Up-DEG |
| LINC00342 | 5.06E-119 | 1.2355755 | 0.324 | 0.057 | 1.25E-114 | Up-DEG |
| SCGB3A2 | 5.46E-119 | 2.40216409 | 0.655 | 0.333 | 1.34E-114 | Up-DEG |
| IL32 | 4.23E-98 | 1.24922807 | 0.378 | 0.112 | 1.04E-93 | Up-DEG |
| ATP13A4-AS1 | 1.04E-97 | 1.36744451 | 0.307 | 0.072 | 2.55E-93 | Up-DEG |
| CD55 | 3.12E-81 | 1.37537242 | 0.805 | 0.634 | 7.67E-77 | Up-DEG |
| ZFP36L1 | 1.18E-79 | 1.05316537 | 0.875 | 0.754 | 2.91E-75 | Up-DEG |
| AGR3 | 9.53E-65 | 1.1943592 | 0.538 | 0.325 | 2.35E-60 | Up-DEG |
| MMP7 | 3.31E-45 | 1.60839348 | 0.313 | 0.241 | 8.14E-41 | Up-DEG |
| SCGB3A1 | 4.07E-44 | 2.32648192 | 0.756 | 0.671 | 1.00E-39 | Up-DEG |
| SPINK5 | 3.38E-39 | 1.00077548 | 0.245 | 0.11 | 8.32E-35 | Up-DEG |
| C11orf96 | 0 | -2.08112379 | 0.245 | 0.709 | 0 | Down-DEG |
| CD36 | 0 | -1.38176762 | 0.013 | 0.509 | 0 | Down-DEG |
| EGR1 | 0 | -1.72316924 | 0.593 | 0.893 | 0 | Down-DEG |
| FABP5 | 0 | -2.09014635 | 0.297 | 0.751 | 0 | Down-DEG |
| HHIP | 0 | -2.0274098 | 0.009 | 0.609 | 0 | Down-DEG |
| SFTPC | 0 | -2.57143903 | 0.609 | 0.957 | 0 | Down-DEG |
| SLPI | 0 | -1.95089578 | 0.784 | 0.886 | 0 | Down-DEG |
| DBI | 2.39022042538953e-318 | -1.20789139 | 0.769 | 0.78 | 5.88400562118141e-314 | Down-DEG |
| PEBP4 | 5.13E-304 | -1.3715568 | 0.336 | 0.681 | 1.26E-299 | Down-DEG |
| AFF3 | 1.46E-251 | -1.37605122 | 0.094 | 0.505 | 3.59E-247 | Down-DEG |
| IER2 | 1.46E-248 | -1.28635634 | 0.745 | 0.896 | 3.60E-244 | Down-DEG |
| IRX3 | 2.39E-246 | -1.21426615 | 0.41 | 0.674 | 5.87E-242 | Down-DEG |
| NNMT | 1.16E-242 | -1.8106245 | 0.077 | 0.489 | 2.86E-238 | Down-DEG |
| CA2 | 4.66E-242 | -1.37521305 | 0.184 | 0.6 | 1.15E-237 | Down-DEG |
| TTN | 1.32E-236 | -2.20215517 | 0.076 | 0.472 | 3.26E-232 | Down-DEG |
| PGC | 2.64E-236 | -1.65700414 | 0.372 | 0.726 | 6.49E-232 | Down-DEG |
| ID4 | 2.06E-235 | -1.49392893 | 0.309 | 0.64 | 5.08E-231 | Down-DEG |
| ZDHHC3 | 1.12E-232 | -1.30248194 | 0.306 | 0.595 | 2.75E-228 | Down-DEG |

AT2, alveolar type 2; CAS, cancer-associated secretory.

Table S4. Differential expressed gene list between CAS cells and Club cells

| gene | p_val | avg_log2FC | pct.1 | pct.2 | p_val_adj | DEG |
| --- | --- | --- | --- | --- | --- | --- |
| CEACAM5 | 2.66E-109 | 7.06793874 | 0.588 | 0.025 | 6.55E-105 | Up-DEG |
| TM4SF18 | 4.60E-35 | 7.03407672 | 0.211 | 0 | 1.13E-30 | Up-DEG |
| LRRN4 | 1.12E-35 | 6.84256637 | 0.214 | 0 | 2.77E-31 | Up-DEG |
| CXCL14 | 2.05E-42 | 6.76831654 | 0.287 | 0.008 | 5.04E-38 | Up-DEG |
| ATP13A4-AS1 | 9.82E-47 | 6.63698801 | 0.307 | 0.008 | 2.42E-42 | Up-DEG |
| SPINK1 | 9.40E-39 | 6.61012932 | 0.338 | 0.048 | 2.31E-34 | Up-DEG |
| ACHE | 1.50E-37 | 6.09134118 | 0.251 | 0.006 | 3.69E-33 | Up-DEG |
| MXRA8 | 2.05E-58 | 6.03808049 | 0.359 | 0.006 | 5.05E-54 | Up-DEG |
| QPRT | 3.03E-33 | 5.9386952 | 0.233 | 0.008 | 7.45E-29 | Up-DEG |
| ALOX15B | 8.96E-45 | 5.80768824 | 0.311 | 0.014 | 2.21E-40 | Up-DEG |
| AFAP1-AS1 | 1.90E-32 | 5.78009492 | 0.222 | 0.006 | 4.68E-28 | Up-DEG |
| C19orf81 | 1.39E-30 | 5.61100371 | 0.203 | 0.003 | 3.43E-26 | Up-DEG |
| HSPB8 | 1.15E-36 | 5.47030567 | 0.266 | 0.014 | 2.83E-32 | Up-DEG |
| MUC3A | 1.54E-62 | 5.42937923 | 0.378 | 0.006 | 3.79E-58 | Up-DEG |
| COL6A2 | 3.42E-32 | 5.36861166 | 0.229 | 0.008 | 8.42E-28 | Up-DEG |
| GJA1 | 6.19E-37 | 5.26513334 | 0.271 | 0.014 | 1.52E-32 | Up-DEG |
| NAPSA | 1.53E-300 | 5.23656791 | 0.963 | 0.153 | 3.76E-296 | Up-DEG |
| SEZ6L2 | 3.03E-70 | 5.2159803 | 0.476 | 0.042 | 7.45E-66 | Up-DEG |
| SLC16A4 | 1.42E-30 | 5.16628466 | 0.226 | 0.011 | 3.49E-26 | Up-DEG |
| GGTLC1 | 1.92E-69 | 5.10126148 | 0.443 | 0.02 | 4.73E-65 | Up-DEG |
| HOXD1 | 3.54E-36 | 5.05374595 | 0.26 | 0.011 | 8.72E-32 | Up-DEG |
| NELL1 | 4.29E-38 | 4.9919269 | 0.271 | 0.011 | 1.05E-33 | Up-DEG |
| B3GNT8 | 3.49E-46 | 4.90944643 | 0.315 | 0.011 | 8.59E-42 | Up-DEG |
| CLDN18 | 3.65E-47 | 4.89158975 | 0.345 | 0.023 | 8.98E-43 | Up-DEG |
| HPGD | 1.26E-53 | 4.76764768 | 0.416 | 0.045 | 3.11E-49 | Up-DEG |
| CDKL2 | 1.10E-60 | 4.63604043 | 0.417 | 0.025 | 2.70E-56 | Up-DEG |
| HSD17B6 | 8.88E-84 | 4.54522237 | 0.539 | 0.04 | 2.18E-79 | Up-DEG |
| ADAMTS1 | 2.69E-30 | 4.5020725 | 0.242 | 0.017 | 6.63E-26 | Up-DEG |
| ZNF385B | 1.31E-48 | 4.4734114 | 0.347 | 0.02 | 3.23E-44 | Up-DEG |
| P3H2 | 6.14E-45 | 4.41375425 | 0.336 | 0.023 | 1.51E-40 | Up-DEG |
| PLTP | 2.64E-29 | 4.40194695 | 0.248 | 0.025 | 6.50E-25 | Up-DEG |
| SFTPC | 9.83E-49 | 4.39432632 | 0.609 | 0.601 | 2.42E-44 | Up-DEG |
| SCTR | 9.71E-60 | 4.3806402 | 0.407 | 0.023 | 2.39E-55 | Up-DEG |
| MAP1B | 8.27E-30 | 4.27047772 | 0.249 | 0.023 | 2.04E-25 | Up-DEG |
| BOK | 5.90E-40 | 4.21812432 | 0.327 | 0.034 | 1.45E-35 | Up-DEG |
| PGC | 1.05E-48 | 4.20879312 | 0.372 | 0.031 | 2.59E-44 | Up-DEG |
| BCAT1 | 1.74E-38 | 4.19325429 | 0.294 | 0.02 | 4.29E-34 | Up-DEG |
| DUXAP8 | 2.47E-28 | 4.19004107 | 0.209 | 0.008 | 6.09E-24 | Up-DEG |
| SORBS1 | 2.95E-42 | 4.10102554 | 0.309 | 0.017 | 7.26E-38 | Up-DEG |
| NQO1 | 3.88E-47 | 4.08953289 | 0.441 | 0.105 | 9.55E-43 | Up-DEG |
| CADM1 | 2.58E-78 | 4.02635452 | 0.548 | 0.062 | 6.35E-74 | Up-DEG |
| ETV4 | 7.52E-26 | 4.02270435 | 0.202 | 0.011 | 1.85E-21 | Up-DEG |
| EGFL7 | 5.05E-25 | 3.98764392 | 0.217 | 0.02 | 1.24E-20 | Up-DEG |
| C1orf167 | 3.23E-28 | 3.97013925 | 0.216 | 0.011 | 7.96E-24 | Up-DEG |
| NRARP | 1.19E-78 | -4.0035717 | 0.055 | 0.439 | 2.93E-74 | Down-DEG |
| IL33 | 6.34E-64 | -4.0127957 | 0.02 | 0.306 | 1.56E-59 | Down-DEG |
| SOX2 | 1.35E-106 | -4.0998524 | 0.054 | 0.521 | 3.33E-102 | Down-DEG |
| TNFAIP2 | 8.71E-100 | -4.2336903 | 0.101 | 0.575 | 2.14E-95 | Down-DEG |
| NTN1 | 2.69E-63 | -4.2383403 | 0.022 | 0.309 | 6.61E-59 | Down-DEG |
| CXCL8 | 5.33E-98 | -4.3077297 | 0.11 | 0.544 | 1.31E-93 | Down-DEG |
| S100P | 1.46E-70 | -4.3300222 | 0.085 | 0.405 | 3.59E-66 | Down-DEG |
| WNK2 | 4.28E-56 | -4.4004152 | 0.024 | 0.286 | 1.05E-51 | Down-DEG |
| MIR205HG | 8.12E-85 | -4.4146907 | 0.014 | 0.346 | 2.00E-80 | Down-DEG |
| IGFBP3 | 3.03E-60 | -4.415112 | 0.034 | 0.32 | 7.46E-56 | Down-DEG |
| PAX9 | 4.39E-48 | -4.5320224 | 0.011 | 0.218 | 1.08E-43 | Down-DEG |
| KRT4 | 6.80E-63 | -4.5995811 | 0.041 | 0.331 | 1.67E-58 | Down-DEG |
| HSD17B13 | 6.43E-49 | -4.6800014 | 0.012 | 0.221 | 1.58E-44 | Down-DEG |
| ADAM28 | 1.98E-108 | -4.7052216 | 0.042 | 0.501 | 4.88E-104 | Down-DEG |
| EYA2 | 1.17E-69 | -4.7346974 | 0.003 | 0.232 | 2.87E-65 | Down-DEG |
| KRT17 | 1.31E-65 | -4.7349966 | 0.033 | 0.334 | 3.23E-61 | Down-DEG |
| KLK10 | 4.46E-124 | -4.7391358 | 0.054 | 0.558 | 1.10E-119 | Down-DEG |
| RHOV | 1.10E-143 | -4.7840505 | 0.052 | 0.601 | 2.72E-139 | Down-DEG |
| CCNO | 3.20E-144 | -4.7996309 | 0.06 | 0.62 | 7.87E-140 | Down-DEG |
| CXCL1 | 3.24E-172 | -4.9642118 | 0.081 | 0.717 | 7.97E-168 | Down-DEG |
| PPP1R1A | 3.94E-55 | -5.0312148 | 0.008 | 0.227 | 9.69E-51 | Down-DEG |
| LCN2 | 1.49E-215 | -5.0391993 | 0.261 | 0.776 | 3.66E-211 | Down-DEG |
| TCF4 | 7.73E-88 | -5.1711513 | 0.015 | 0.36 | 1.90E-83 | Down-DEG |
| IRAK3 | 1.27E-53 | -5.216405 | 0.011 | 0.235 | 3.12E-49 | Down-DEG |
| SERPINF1 | 1.28E-140 | -5.3314967 | 0.02 | 0.524 | 3.15E-136 | Down-DEG |
| PTGFR | 1.25E-93 | -5.3810441 | 0.006 | 0.331 | 3.09E-89 | Down-DEG |
| MUC16 | 1.65E-73 | -5.3908132 | 0.014 | 0.312 | 4.07E-69 | Down-DEG |
| TGM2 | 1.70E-124 | -5.5176846 | 0.027 | 0.49 | 4.18E-120 | Down-DEG |
| KLK13 | 9.69E-87 | -5.5606261 | 0.022 | 0.371 | 2.39E-82 | Down-DEG |
| PI3 | 9.91E-75 | -5.8268843 | 0.003 | 0.252 | 2.44E-70 | Down-DEG |
| SAA2 | 1.84E-92 | -5.8581254 | 0.002 | 0.295 | 4.52E-88 | Down-DEG |
| KYNU | 1.15E-58 | -6.0230286 | 0.006 | 0.224 | 2.84E-54 | Down-DEG |
| SAA1 | 6.28E-115 | -6.0977044 | 0.004 | 0.377 | 1.55E-110 | Down-DEG |
| HCAR2 | 2.12E-96 | -6.2540228 | 0.006 | 0.34 | 5.22E-92 | Down-DEG |
| FAM3D | 4.95E-97 | -6.6057853 | 0.007 | 0.343 | 1.22E-92 | Down-DEG |
| ZG16B | 1.11E-74 | -6.813383 | 0.002 | 0.249 | 2.72E-70 | Down-DEG |
| RARRES1 | 4.51E-137 | -6.8669288 | 0.012 | 0.473 | 1.11E-132 | Down-DEG |
| KLK14 | 2.11E-84 | -6.964464 | 0.001 | 0.263 | 5.20E-80 | Down-DEG |
| CHST9 | 1.91E-188 | -6.9749101 | 0.006 | 0.581 | 4.70E-184 | Down-DEG |
| PTPRZ1 | 6.88E-59 | -7.040358 | 0.003 | 0.21 | 1.69E-54 | Down-DEG |
| ATP12A | 5.69E-103 | -7.257722 | 0.003 | 0.34 | 1.40E-98 | Down-DEG |
| TMEM45A | 1.68E-229 | -7.4123618 | 0.016 | 0.686 | 4.13E-225 | Down-DEG |
| CXCL6 | 1.58E-137 | -7.4326908 | 0.007 | 0.453 | 3.90E-133 | Down-DEG |
| VMO1 | 7.05E-156 | -7.4379904 | 0.018 | 0.533 | 1.74E-151 | Down-DEG |
| MUC5B | 1.18E-161 | -7.6492991 | 0.02 | 0.555 | 2.89E-157 | Down-DEG |
| PSCA | 2.94E-85 | -7.7249151 | 0.004 | 0.292 | 7.24E-81 | Down-DEG |
| AC007681.1 | 1.56E-102 | -7.7531342 | 0.003 | 0.337 | 3.84E-98 | Down-DEG |
| LTF | 1.09E-156 | -8.4824323 | 0.007 | 0.501 | 2.69E-152 | Down-DEG |
| CYP2F1 | 1.48E-245 | -9.2108787 | 0.003 | 0.686 | 3.63E-241 | Down-DEG |
| CHP2 | 4.25E-68 | -9.2133266 | 0 | 0.204 | 1.05E-63 | Down-DEG |
| CLCA2 | 2.68E-75 | -9.4236945 | 0.001 | 0.238 | 6.59E-71 | Down-DEG |
| SCGB1A1 | 0 | -9.6565912 | 0.164 | 0.926 | 0 | Down-DEG |
| BPIFA1 | 1.90E-56 | -9.7894063 | 0.004 | 0.201 | 4.67E-52 | Down-DEG |
| SERPINB3 | 1.25E-77 | -11.209902 | 0 | 0.241 | 3.08E-73 | Down-DEG |
| BPIFB1 | 7.35E-268 | -11.33262 | 0.009 | 0.734 | 1.81E-263 | Down-DEG |
| MSMB | 9.38E-100 | -11.408066 | 0.002 | 0.314 | 2.31E-95 | Down-DEG |

CAS, cancer-associated secretory.
